# YAMP: a containerized workflow enabling reproducibility in metagenomics research

**DOI:** 10.1093/gigascience/giy072

**Published:** 2018-06-18

**Authors:** Alessia Visconti, Tiphaine C Martin, Mario Falchi

**Affiliations:** Department of Twin Research and Genetic Epidemiology, King’s College London, Westminster Bridge Road, SE1 7EH, London, UK

**Keywords:** metagenomics, reproducibility, workflow, containerization, docker, singularity

## Abstract

YAMP ("Yet Another Metagenomics Pipeline") is a user-friendly workflow that enables the analysis of whole shotgun metagenomic data while using containerization to ensure computational reproducibility and facilitate collaborative research. YAMP can be executed on any UNIX-like system and offers seamless support for multiple job schedulers as well as for the Amazon AWS cloud. Although YAMP was developed to be ready to use by nonexperts, bioinformaticians will appreciate its flexibility, modularization, and simple customization.

## Background

Thanks to the increased cost-effectiveness of high-throughput technologies, the number of studies collecting and analyzing large amounts of data has surged, opening new challenges for data analysis and research reproducibility. A ubiquitous lack of repeatability and reproducibility has, in fact, been observed, and a recent *Nature* survey of 1,576 researchers showed that more than 50% and 70% failed to reproduce their own and other scientists’ experiments, respectively [1]. Unavailability of primary data and computational experimentation have been named as the major culprits for this reproducibility crisis, with many studies relying on *ad hoc* scripts and not publishing the necessary code and/nor sufficient details to reproduce the reported results [2–4]. In addition, variations across workstations and operating systems represent another obstacle [5, 6]. To overcome this issue, tools that allow the development of workflows [7] and software containers [8] have been proposed [9]. In fact, containerized, well-structured workflows allow storage of every detail of the workflow execution, including the software’s versions and parameters (*provenance* [10]), and nullify system variations [6], therefore, guaranteeing a study’s repeatability and reproducibility. Containerized workflows also facilitate collaborative projects by ensuring identical analysis processes, thus, comparable results, and allow the automatization of data-intensive repetitive tasks [11]. Moreover, they save users with little bioinformatics or computational expertise from the hassles of installing the required pieces of software and of designing and implementing often complex analysis orchestrations, while expert bioinformaticians can use them as a starting point for customized analyses, thus avoiding redundant solutions.

In metagenomics research, several analysis pipelines have been developed. However, they either do not support containerization (e.g., MetAMOS [12], MOCAT2 [13], RAMMCAP [14]), thus, potentially compromising reproducibility, or they require users to upload their unpublished and/or confidential data on third-party servers (e.g., IMG/M [15], the EBI metagenomics pipeline [16], MG-RAST [17]). Based on the available resources, they can spend several days on these servers waiting to be processed [18], with data privacy concerns for some researchers [19]. Scalable metagenomic pipelines that allow both local and cloud execution have been proposed, such as CloVR-Metagenomics [20], and those implemented using the Galaxy platform [21, 22]. However, the former lacks steps for quality control (QC) and allows processing reads only generated with the Roche 454 pyrosequencing platform [23], and the latter requires nontrivial expertise for local installation [24], with porting issues observed among different Galaxy versions [25]. QC is also often overlooked. For instance, both MetAMOS and the EBI metagenomics pipeline do not include a step for removing contaminant genomes, with the latter also not discarding identical duplicates. Ignoring decontamination may lead to reads not belonging to the studied ecosystem to be used in downstream analyses, causing potential mismapping on reference databases and, therefore, erroneous functional profiling, especially in low-biomass environments [26]. Moreover, the presence of contaminating human reads raises privacy concerns. There are now two studies able to mine and exploit hosts’ genetic material from publicly available metagenomics samples [26, 27]. Retaining duplicated reads, usually considered as technical artifacts derived from polymerase chain reaction (PCR) amplification [28], may hamper the correct estimation of both community composition and functional capabilities [29]. Finally, MG-RAST performs de-duplication after quality trimming, potentially introducing biases due to the fact that trimming, by modifying the read sequence, may mask true duplicates or generate false ones.

Here, we present “Yet Another Metagenomics Pipeline” (YAMP), a ready-to-use containerized workflow that, using state-of-the-art tools, processes raw shotgun metagenomics sequencing data up to the taxonomic and functional annotation. YAMP is implemented in Nextflow [6] and is accompanied by a Docker [30] and a Singularity [31] container. The YAMP script, parameters, and documentation are available at https://github.com/alesssia/YAMP.

### The YAMP workflow

**Figure 1:**
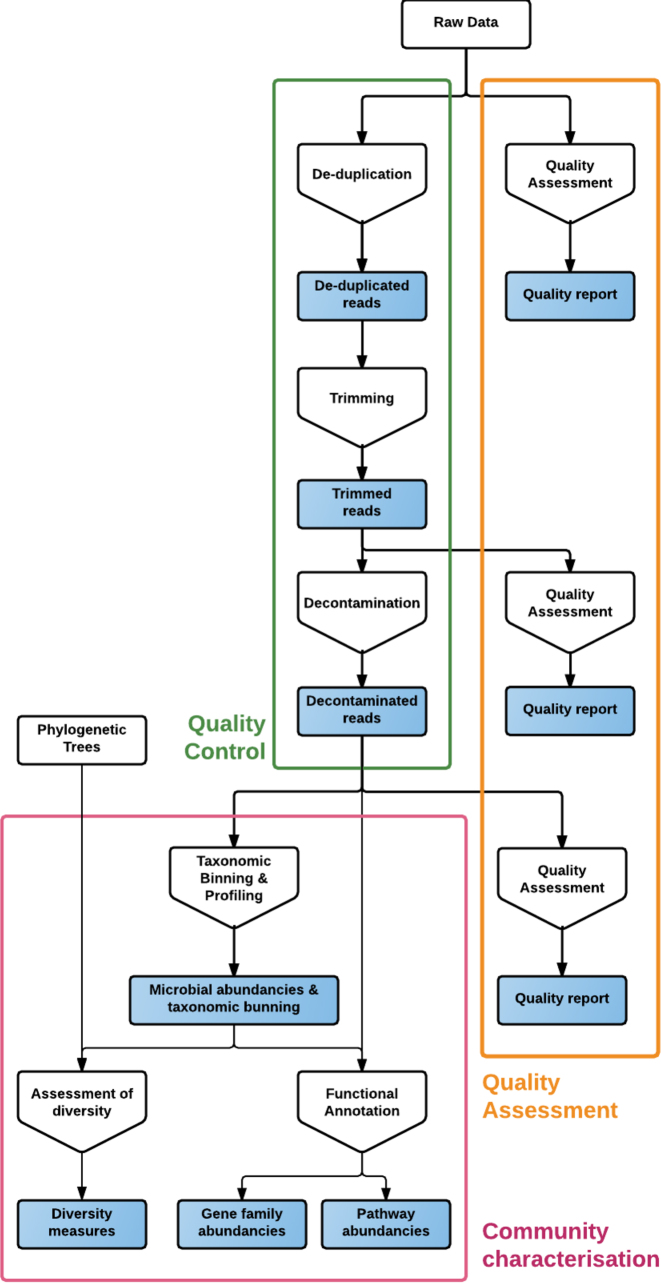
The YAMP workflow. White rectangles represent data to be provided as input, and blue rectangles those produced in output. Pentagons represent the analysis steps.

The YAMP workflow is composed of three analysis blocks: QC (Fig.1, green rectangle) complemented by several steps of assessment and visualization of data quality (Fig.1, orange rectangle) and community characterization (Fig.1, pink rectangle).

The QC starts with an optional step of de-duplication, where identical reads, potentially generated by PCR amplification, are removed. The optionality of this step allows retaining natural duplicates when PCR-free library preparation approaches (e.g., TruSeq) are used. Next, reads are filtered to remove adapters, known artifacts, and phiX, and then quality trimmed. Notably, YAMP removes duplicates before trimming, avoiding the introduction of biases due to the reads’ sequence modifications. Reads that become too short after trimming are discarded. Indeed, they may map to multiple genomes or genomic regions and compromise downstream analyses. When paired-end reads are at hand, singleton reads (i.e., paired-end reads whose mates have been removed) are preserved in order to retain as much information as possible. Finally, reads are screened for contaminants, e.g., reads that do not belong to the studied ecosystem. It should be kept in mind when preparing the custom database of contaminant reads, that many low-complexity sequences and certain features (e.g., ribosomes) are highly conserved among species and should be removed to avoid false-positive matches. The implemented QC is accompanied by multiple steps that assess and visualize the reads’ quality in order to evaluate the quality of the raw data and the effectiveness of the trimming and decontamination step. QC is followed by multiple steps aimed at estimating multiple α-diversity measures and at characterizing the taxonomic and functional profiles of the microbial community, i.e., identifying and quantifying the microorganisms present in the metagenomic sample (taxonomic binning and profiling) and their functional capabilities (functional characterization).

### Implementation

YAMP is developed in Nextflow, a workflow management system that allows the effortless development, deployment, and execution of complex distributed computational workflows [6]; it has been used in several life-science projects (e.g.,[32–34]). Nextflow allows for user-transparent high-level parallelization and offers out-of-the-box support for distributed computational environments, ensuring the scalability of large projects. Its executor allows porting of workflows on any UNIX-based system (e.g., local machine, high-performance computing [HPC] facilities) in a seamless fashion. Reproducibility is guaranteed by a user-transparent integration with Docker and Singularity and with the BitBucket [35], GitHub [36], and GitLab [37] code repositories, therefore, ensuring consistent tracking of both software and code version. The so-called retrospective provenance, i.e., the description of each completed analysis step along with details about its underlying execution environment [10], is captured by task execution reports that record, among the others, the exact command executed, the tasks’ working directory, the environment and output, as well as the container image.

YAMP is accompanied by a Docker [30] and a Singularity [31] container. Docker defines a platform-independent, virtualized, lightweight operating system that includes all the pieces of software required by YAMP and traces their versioning. Singularity allows these features to be transferred to HPC systems, with which Docker is inherently incompatible. Along with this single-container approach, YAMP also supports a multicontainer scenario. Indeed, while the former is easier to manage for users with limited computational experience and allows a more agile deployment, the latter makes YAMP customization possible without losing the advantages of a containerized solution in terms of reproducibility and ease of setup.

YAMP integrates state-of-the-art tools for the analysis of metagenomic data. QC is performed with a number of tools that belong to the BBmap suite [38], namely, clumpify, BBduk, and BBwrap, which are well established and allow processing both single- and paired-end reads from all the major sequencing platforms (i.e., Illumina, Roche 454 pyrosequencing, Sanger, Ion Torrent, Pacific Biosciences, and Oxford Nanopore). They are also computationally efficient, thus, scalable to large metagenomics projects and samples. FastQC [39], which provides very detailed reports on reads’ quality, is used to perform QC assessment and visualization. Taxonomic binning and profiling is performed with MetaPhlAn2 [40], which uses clade-specific markers to both detect the microorganisms and to estimate their relative abundance. The clade-based approach implemented in MetaPhlAn2 has been shown to scale to large datasets and was found to be effective in quantitatively profiling the microbial composition during the Human Microbiome Project (HMP) [41] and the Critical Assessment of Metagenome Interpretation challenge [42]. The functional capabilities of the microbial community are assessed by the HUMAnN2 pipeline [43], an extension of the pipeline originally developed by the HMP Metabolic Reconstruction Working Group to infer the functional and metabolic potential of microbial communities during the HMP [44]. Briefly, the HUMAnN2 pipeline first stratifies the community in known and unclassified organisms using the MetaPhlAn2 results and the ChocoPhlAn pan-genome database. It then combines these results with those obtained through an organism-agnostic search on the UniRef proteomic database and on the MetaCyc database of metabolic pathways and enzymes [45]. The identified taxonomic profile is also used by YAMP to evaluate multiple α-diversity measures through the alpha_diversity.py function available in the widely used QIIME pipeline [46]. QIIME is an extremely modular and efficient pipeline designed for the analysis of amplicon (e.g., 16S or 18S rRNA genes) sequencing data, which implements a number of functions to investigate ecological features relevant also to metagenomics research.

### YAMP Input/Output

YAMP accepts in input both single- and paired-end FASTQ files. Users can customize the workflow execution either by using command line options or by modifying a simple plain-text configuration file, where parameters are set as key-value pairs. While the parameters should be tuned according to the dataset at hand, to assist nonexpert users in their analyses of human metagenomics data, we provide a set of default parameters derived from our own analysis experience. We suggest retaining bases with a Phred score of at least 10 (Q10), representing a base call accuracy of 90%, i.e., the probability of calling a base out of 10 incorrectly. This allows the retrieval of low-coverage regions, therefore, improving the total genome recovery and contiguity, an aspect of utmost importance when the QC'ed reads are used for assembly. We also recommend discarding all reads shorter than 60 bp after trimming, corresponding to a complexity of 4^60^ or less. This length is considered the lowest for avoiding spurious signals when carrying on functional characterization via HUMAnN2 [47–49]. Next, we propose using a minimum alignment identity of 95% (maximum indel length, 3 bp) to identify contaminant reads, which has been shown to possibly zero the number of false positives when used on opportunely created custom databases of contaminant reads [50]. Finally, we suggest using the UniRef90 protein database, as its clusters are more likely to be iso-functional and nonredundant. However, the UniRef50 protein database is preferred when dealing with poorly characterized microbiomes.

**Figure 2:**
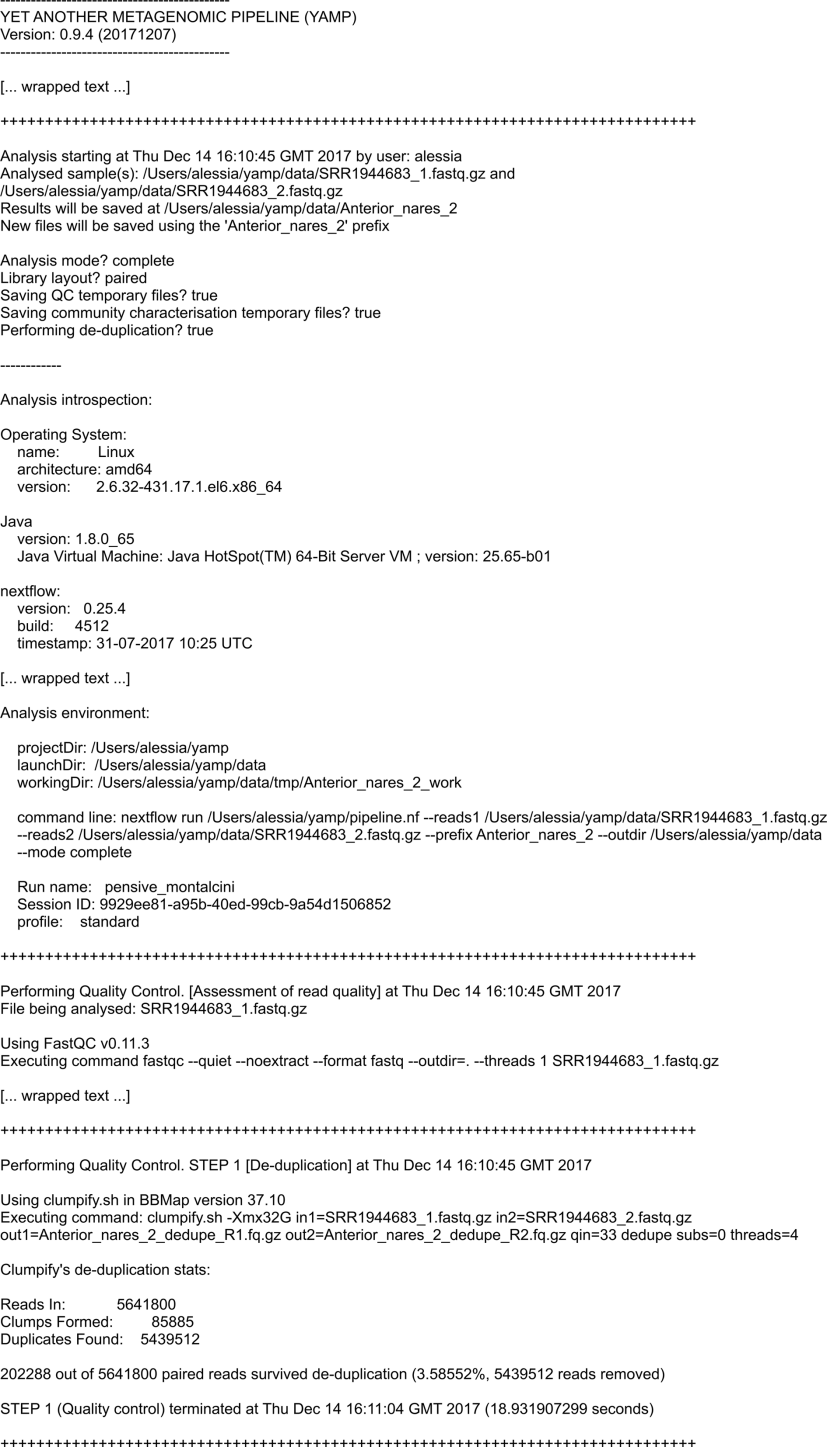
An excerpt from the YAMP execution log.

**Figure 3:**
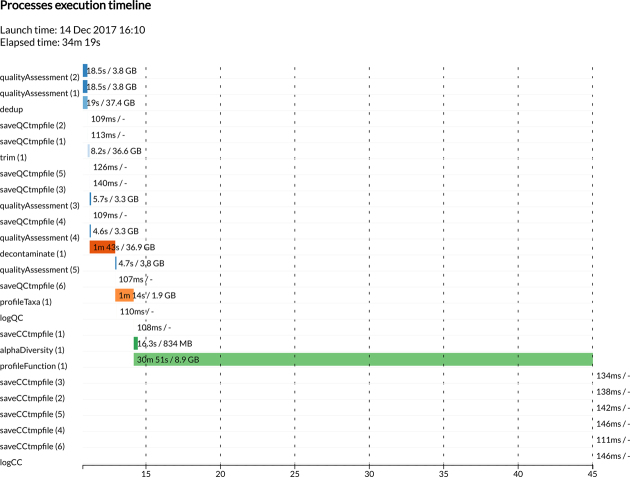
An example of the YAMP execution profile. YAMP returns the time spent during its complete execution and in each step, as well as the steps’ memory peaks.

The output generated by YAMP includes a FASTQ file of QC’ed reads, the taxonomy composition along with relative abundance of microbes, genes and pathways, the pathways coverage, and multiple α-diversity measures. An option allows users to retain temporary files, such as those generated by the QC steps or during the HUMAnN2 execution. Additionally, YAMP outputs several QC reports; a very detailed log file recording information about each analysis step, which ensures the retrospective provenance (Fig.2); and statistics of memory usage and time of execution (Fig.3). It should be noted that the disk space required by the files generated by YAMP is, on average, about seven times the size of the raw data files. Particular attention should be paid when multiple samples are processed simultaneously, as in multicore machines or HPC facilities. However, discarding temporary files, as we suggest, will require a final disk space of 20%–70% the size of the raw data files, with higher-quality files requiring more space due to the small number of reads discarded during the QC process.

### Results

To compare YAMP to existing metagenomic analysis workflows, we simulated datasets that included either different percentages of human contamination or of artificial duplicates. Then, to facilitate the discussion on YAMP computational requirements and to assess its ability to reproduce research results described in the literature, we carried out a real-world case study, which included 18 samples collected from different body sites. Notably, despite both the simulation and the real-world case study focus on human metagenomic data, YAMP can be used for the analysis of data that originate from virtually any environment.

#### Simulation study

In the first simulated scenario, we aimed at testing the impact of contaminant reads on the functional characterization of the microbial community. Therefore, we generated 5 metagenomic samples simulating a human oral community with 13 bacterial species and including a variable amount of human reads as contaminants. The relative proportion of the bacterial genomes followed that suggested by Zhou et al. [51] (Supplementary Table S1). The percentage of contaminant reads was 1%, 5%, 25%, 50%, and 80%, which is in line with the amounts observed in the literature for human samples [26, 52, 53]. For instance, the HMP Consortium targeted 49% of the total reads as human and also observed that samples collected from soft tissue and preparations from saliva showed the highest human contamination, with samples from mid-vagina, saliva, anterior nares, and throat including 96%, 80%, 82%, and 75% of human DNA sequence, respectively. Stool was only marginally affected, including less than 1% of human contamination [53]. We compared the functional profiles inferred by YAMP with those generated using the EBI metagenomics pipeline (version 4.1), which does not include a decontamination step. These two workflows use different databases for the functional characterization (i.e., MetaCyc and both the InterPro and the Gene Ontology (GO) databases, respectively), and their results could not be directly compared. Therefore, for both tools, we evaluated the root-mean-square error (RMSE) between the functional profiles inferred for each of the contaminated dataset and that inferred for a baseline dataset with no human contamination (the lower the RMSE, the better the fit). YAMP showed the best performances, with an RMSE <1.25 × 10^−6^ regardless of the amount of human contamination (Table 1, Supplementary Fig. S1). Notably, despite the fact that YAMP was not able to remove all the contaminant reads (likely due to the masking of the low-complexity and highly conserved region in the reference genome used during the decontamination step), its performances are stable, plausibly thanks to the high specificity of the annotation database used. The EBI metagenomics pipeline guaranteed appreciable results; the maximum RMSE was 0.086 with 80% human contamination and using functional annotations from the GO Slim database. However, while its GO-based results showed errors that were uniformly distributed along the identified annotations and that increased with the level of human contamination, results on the InterPro database seemed to be concentrated on a few specific domains (Supplementary Fig. S1). Three of these domains (*“L1 transposable element, dsRBD-like domain”; “L1 transposable element, trimerization domain”;*and *“Domain of unknown function DUF1725”*), which were not detected in the baseline dataset, are connected to the long interspersed nuclear element 1 (L1), an active retrotransposon that comprises approximately 17% of the human genome [54], which is an obvious sign of uncontrolled contamination.

**Table 1:** Results of the first simulation study (human contamination)

			YAMP		The EBI metagenomics pipeline		
	${N_{{human}}}$	${N_{{total}}}$	${N_{{detected}}}$	${RMSE}_{{MetaCyc}}$	${RMSE}_{{GO}}$	${RMSE}_{{GO Slim}}$	${RMSE}_{{InterPro}}$
1%	6,630	662,947	4,712	9.24 × 10^−7^	2.01 × 10^−4^	4.45 × 10^−4^	2.99 × 10^−4^
5%	34,543	690,860	24,558	9.88 × 10^−7^	9.16 × 10^−4^	2.12 × 10^−3^	1.15 × 10^−3^
25%	218,772	875,089	155,474	8.82 × 10^−7^	3.05 × 10^−3^	8.98 × 10^−3^	5.28 × 10^−3^
50%	656,317	1,312,634	465,920	9.94 × 10^−7^	7.78 × 10^−3^	0.023	0.018
80%	2,625,268	3,281,585	1,864,367	1.25 × 10^−6^	0.028	0.086	0.059

For each level of human contamination, we report the number of human and total reads and, only for YAMP, we report the number of human reads correctly detected and removed. RMSE values were evaluated on the inferred proportions using a dataset with no human contamination as a baseline. For the EBI metagenomics pipeline (v4.1), we report RMSE values evaluated on the three databases used for functional characterization.

In the second simulated scenario, we aimed at testing the impact of artificial duplicates on the microbial community’s functional characterization. Therefore, we generated three datasets simulating the human oral community described previously (Supplementary Table S1) but without any natural duplicates and introducing percentages of duplication of about 0.25%, 1.25%, and 5%, which is in line with the values described in the literature [55, 56]. Consistent with the observations that GC-rich DNA sequences are difficult to amplify and that the lower the CG content the higher the probability of an amplification bias [55], we allowed the introduction of duplicates, mostly from bacteria, with the lowest GC content, namely, *Streptococcus peroris, Veillonella atypica, Veillonella parvula*, and *Veillonella dispair* (see Methods). We compared the functional profiles generated by YAMP with those generated using the EBI metagenomics pipeline (version 4.1), which does not include a de-duplication step, and with those generated using the MG-RAST pipeline (version 4.0.3), which performs trimming before de-duplication, potentially introducing biases. As in the previous simulation study, we assessed their performances on a baseline dataset that, in this case, did not include any duplicate. YAMP was again the best performer, with an RMSE <1.17 × 10^−6^ regardless of the amount of duplication and with stable results at each duplication level (Table 2, Supplementary Fig. S2). The EBI metagenomics pipeline offered excellent results when evaluated over the InterPro database annotation (RMSE <6.85 × 10^−3^). However, its performance slightly degraded when the evaluation was performed on the GO and GO Slim annotations, where we observed, at a 5% level of duplication, a maximum RMSE of 0.011 and 0.022, respectively (Table 2). MG-RAST allows functional characterization to be performed using several databases (e.g., RefSeq, GenBank, SEED Subsystems, Kyoto Encyclopedia of Genes and Genomes [KEGG]). For the sake of simplicity, we used the annotations derived from the SEED Subsystems Ontology [57], which are provided as a precomputed summary of the reads assigned at the highest level of this functional hierarchy, and from the KEGG database, which we preprocessed to extract the number of reads assigned at each KEGG annotation – level 3 (see Methods). MG-RAST performances were acceptable when evaluated on the KEGG database (RMSE <0.017) but decreased sensibly on the SEED Subsystems Ontology (RMSE >0.243, Table 2), mostly due to a progressive overestimation of the "Nucleosides and Nucleotides" ontology term (estimated to be 3.69%, 4.93%, 4.98%, and 5.27% at baseline and at 0.25%, 1.25%, and 5% percentage of duplication, respectively).

**Table 2:** Results of the second simulation study (artificial duplicates)

			YAMP		The EBI metagenomics pipeline			MG-RAST		
	${N_{{duplicates}}}$	${N_{{total}}}$	${N_{{detected}}}$	${RMSE}_{{MetaCyc}}$	${RMSE}_{{GO}}$	${RMSE}_{{GO Slim}}$	${RMSE}_{{InterPro}}$	${N_{{detected}}}$	${RMSE}_{{SEED Subsystems}}$	${RMSE}_{{KEGG}}$
0%	0	640 492	1	–	–	–	–	47,839	–	–
0.25%	1,640	642 132	1,641	1.17 × 10^−6^	1.35 × 10^−3^	3.50 × 10^−3^	7.77 × 10^−4^	49,249	0.243	4.55 × 10^−3^
1.25%	8,206	648 698	8,207	1.07 × 10^−6^	3.66 × 10^−3^	8.98 × 10^−3^	2.32 × 10^−3^	54,875	0.253	7.77 × 10^−3^
5%	32,835	673 327	32,836	9.75 × 10^−7^	0.011	0.022	6.85 × 10^−3^	75,925	0.312	0.017

For each level of artificial duplicates, we report the number of duplicates and total reads and, for YAMP and MG-RAST, we report the number of duplicated reads removed. RMSE values were evaluated on the inferred proportions using a dataset with no duplicated reads as a baseline. For the EBI metagenomics pipeline (v4.1), we report RMSE values evaluated on the three databases used for functional characterization. For MG-RAST (v4.0.3), we report RMSE values evaluated on two of the databases available for functional characterization (i.e., SEED Subsystems and KEGG).

#### Real-world case study

**Figure 4:**
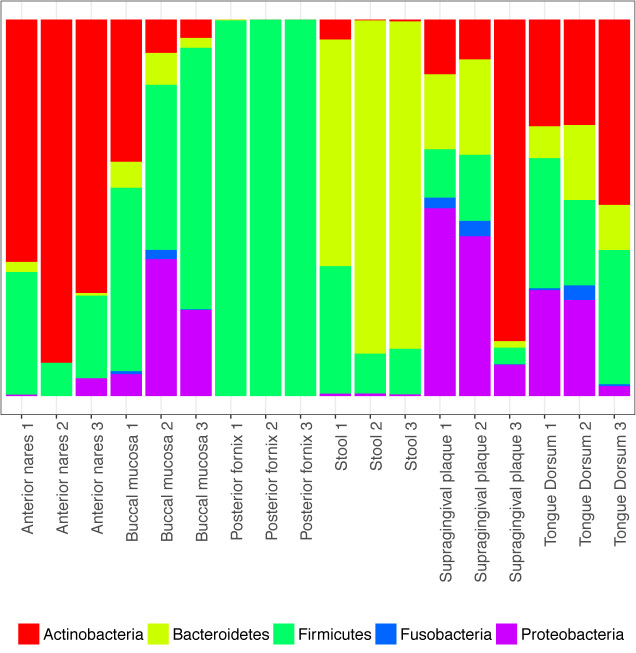
Phylum-level relative abundances. Each vertical bar represents a sample. Phylum relative abundances were estimated by YAMP using MetaPhlAn2. Unspecified viral phyla are not shown.

We analyzed 18 randomly selected samples from six body sites sequenced during phase III of the HMP [53] (Table 3). On average, the selected samples included 12.6 M paired-end reads (25.2 M reads in total), which yielded 13.3 M QC’ed reads (including both paired-end and singleton reads), and were processed in an average time of 2 hours using four threads on a machine sporting a 2.60-GHz Intel Xeon processor with 32 GB of random access memory and using the default YAMP parameters (Table 3). At the phylum level, each body site showed a characteristic signature (Fig. 4), with a predominance of Actinobacteria in the airway, Firmicutes in the vagina, Bacteroidetes in the stool, and a mixture of Actinobacteria, Firmicutes, and Proteobacteria in the oral cavity, as observed in previous studies [58]. A site-specific microbial signature was also present at the species level, where both the principal coordinate analysis (PCoA), evaluated using the Bray-Curtis dissimilarity (Supplementary Fig. S3 and S4), and the hierarchical clustering, computed on the Manhattan distances between species relative abundances (Supplementary Fig. S5), showed that the taxonomy composition was sufficient to discriminate among body sites, even though it had limited ability in distinguishing between different loci in the oral cavity.

**Table 3:** Run accession number and statistics for 18 randomly selected samples from the HMP phase III [53]

Body site	Locus	SRA accession number	Number of raw paired-end reads	Number of QC’ed reads: paired-end; singletons	Running time	Disk space occupation: raw data; processed data with; without temporary files
		SRR1944674	1,181,169	590,714; 42,241	39 m 02 s	107.46 MB; 1.38 GB; 81.82 MB
Airway	Anterior nares	SRR1944683	2,820,900	56,151; 9,513	31 m 31 s	72.96 MB; 314.55 MB; 11.69 MB
		SRR1952439	14,635,701	201,260; 17,345	42 m 00 s	379.16 MB; 1.39 GB; 28.19 MB
						
		SRR1951826	7,956,274	7,121,697; 494,289	2 h 15 m 39 s	1.09 GB; 14.83 GB; 934.41 MB
Gut	Stool	SRR1944873	11,033,130	9,796,817; 942,566	2 h 26 m 01 s	1.55 GB; 18.95 GB; 1.18 GB
		SRR1952058	5,834,232	5,484,362; 248,819	1 h 39 m 10 s	814.33 MB; 10.96 GB; 694.87 MB
						
		SRR1944703	6,231,553	285,906; 24,212	39 m 09 s	184.39 MB; 1.07 GB; 57.11 MB
	Buccal mucosa	SRR1952437	15,361,468	3,451,844; 149,714	1 h 19 m 26 s	800.49 MB; 7.57 GB;405.52 MB
		SRR1952380	11,872,420	631,595; 41,957	49 m 07 s	365.74 MB; 1.15 GB; 84.45 MB
		SRR1952435	16,169,911	13,620,835; 672,610	2 h 44 m 56 s	1.99 GB; 15.42 GB; 1.45 GB
Oral cavity	Supragingival plaque	SRR1952436	21,971,588	17,237,506; 987,950	4 h 07 m 11 s	2.59 GB; 33.35 GB; 1.96 GB
		SRR1952492	19,202,739	8,040,737; 1,805,898	1 h 51 m 05 s	1.80 GB; 16.25 GB; 1.09 GB
		SRR1944869	8,074,428	6,140,295; 499,284	1 h 36 m 58 s	994.07 MB; 12.04 GB; 767.47 MB
	Tongue dorsum	SRR1952378	15,024,409	12,622,724; 891,920	3 h 17 m 30 s	1.93 GB; 25.00 GB; 1.48 GB
		SRR1952379	42,173,063	29,697,754; 2,084,990	7 h 10 m 23 s	5.59 GB; 60.41 GB; 3.04 GB
						
		SRR1951760	10,611,721	373,021; 24,484	42 m 19 s	277.17 MB; 1.57 GB; 44.39 MB
Vagina	Posterior fornix	SRR1944797	8,242,829	120,519; 10,009	35 m 14 s	203.81 MB; 967.59 MB; 17.21 MB
		SRR1944845	8,537,797	140,658; 10,779	34 m 19 s	213.02 MB; 882.72 MB; 30.06 MB

Samples were processed using four threads on a machine sporting a 2.60-GHz Intel Xeon processor with 32 GB of RAM. The required disk space for the processed data does not include the size of the raw data file.

## Discussion

In conclusion, with YAMP, we provide a user-friendly workflow that makes possible the analysis of whole shotgun metagenomics data. By supporting containerization, YAMP allows for computational reproducibility and also enables collaborative studies. In fact, while software versions are described in the Docker/Singularity container, the Nextflow script and configuration file capture all the details needed to fully track each step of data processing, therefore, satisfying the prospective provenance requirements, while the very detailed YAMP log file ensures retrospective provenance. Indeed, to ensure reproducibility, researchers should only provide the YAMP configuration file and a link to the container image. Being based on Nextflow, YAMP runs on any UNIX-like system, provides out-of-the-box support for several job schedulers (e.g., PBS, SGE, SLURM) and for Amazon AWS cloud, and its integration with Docker/Singularity is completely user transparent. Finally, while YAMP has been developed to be ready to use by nonexperts and potentially does not require any software installation or parameter tuning, expert bioinformaticians will value its flexibility and simple customization. In fact, the well-defined YAMP modularization and the usage of standard data formats allow both an easy integration of new analysis steps and a customization of existing ones. This is of particular importance in a fast-developing field such as metagenomics, where the analysis guidelines and tools are not stable yet. With YAMP we provide a workflow to which new analysis modules can be easily added and where tools that become outdated can be effortlessly replaced, therefore, securing its sustainability.

YAMP is made available as a Nextflow script that allows a user-friendly execution via the command line. The source code is available in the YAMP GitHub repository, which includes a wiki with full documentation and several tutorials. The Docker/Singularity image can be downloaded and installed from DockerHub.

## Potential Implications

YAMP has been designed with the specific goals of enabling reproducible metagenomics analyses, facilitating collaborative projects, and helping researchers with limited computational experience who are approaching this field of research. However, we are confident that other areas of research would be aided by a more widespread use of containerized, well-structured workflows. Indeed, as outlined in the Background section, today a lack of reproducibility is ubiquitous. In addition to undermining the credibility of scientific research, it has an economical cost, quantified, for instance, in $28,000,000,000/year for preclinical research [59]. On the other hand, ensuring reproducibility does not come for free. Anecdotal evidence suggests that the time spent on a project may increase by 30%–50% [1] and that reproduction of the analysis of a single computational biology article can require up to 280 hours [60]. YAMP along with other containerized workflows, such as the Integrated Meta-omic Pipeline [61] and Bio-Docklets [62], represent a proof-of-concept that shows a simple way to enable reproducible and collaborative research. We also advocate the sharing of such containerized workflows, which will benefit a wide group of researchers, regardless of their computational experience [11].

## Methods

### Data availability

#### Simulation study

The simulated metagenomic samples were generated using the reference human and microbial genomes downloaded from the National Center for Biotechnology Information (Supplementary Table S1; human genome: build GRCh37.p13). Each microbial genome was processed independently and used as input for the randomreads tool from the BBmap suite [38], which allows generation of random reads in various formats. Oral bacterial single-end sequences were generated using the default parameters and asking for a fixed read length of 100 bp, a Phred score ranging from 6 to 40 (average, 20), and with a probability of mutation (single-nucleotide polymorphism, insertion, deletion, substitution, and N calls) of 0.2 (max 10 blocks of Ns per read). We also assigned scaffolds a random exponential coverage level in order to simulate a metagenomics coverage distribution (metagenome = t) and asked for a fixed genomic coverage (Supplementary Table S1). Human single-end sequences were created using the same parameters used for the microbial genomes and asking for a fixed number of reads (Table 1).

In the second simulated scenario, we first de-duplicated each microbial simulated dataset generated beforehand using clumpify [38]. Then, for each of the four species showing the lowest GC content (*S. peroris, V. atypica, V. parvula, and V. dispair*), we generated sets of duplicated reads by randomly selecting and matching reads and quality scores in order to include 40% of identical duplicates. We then merged the de-duplicated dataset with these sets of duplicated reads to build a new reads pool. Next, from the described pool, we randomly selected fixed numbers of reads that were finally merged with the de-duplicated dataset in order to generate each simulated sample. This allowed for duplicates more likely to belong but not limited to the GC-poor microbial genomes and to have duplicated reads that are more likely but not limited to have different quality scores.

The simulated datasets are available from the European Nucleotide Archive website (study accession numbers PRJEB25791 and PRJEB26333; Supplementary Table S2).

#### Real-world case study

The 18 randomly selected samples used to assess YAMP belong to phase III of the HMP [53] and were downloaded from the European Nucleotide Archive website (study accession number PRJNA275349). Samples were collected from healthy adults residing in the United States at the time of sample collection. After genomic DNA extraction, the metagenomics library was prepared using the NexteraXT library construction protocol. Paired-end metagenomics sequencing was performed on the Illumina HiSeq2000 platform with a read length of 100 bp. Sample accession numbers are listed in Table 3.

### Data analysis

#### Simulation Study

Samples were processed with YAMP using the default parameters, as defined in the published YAMP configuration file, and the databases queried during the YAMP execution were deposited on Zenodo [63]. Unmapped reads were discarded. When analyzing the simulated dataset with the EBI metagenomics pipeline (version 4.1) and MG-RAST (version 4.0.3), the default settings were used. Results from the EBI metagenomics pipeline were downloaded via the web user interface, and the functional profiles were evaluated by transforming the proportion of reads assigned to each function to percentage. When evaluating MG-RAST performances, we used the functional profiles evaluated on the SEED Subsystems Ontology and KEGG databases, identified at a default alignment length of >15 bp, e-value <1 × 10^−5^ and with percent identity >60% (as by MG-RAST default). Data for the SEED Subsystems Ontology, annotated at the highest level of the hierarchy, were downloaded via the web user interface, and the functional profiles were evaluated by transforming to percentage the proportion of reads assigned to each function. Data for the KEGG database were downloaded via the web user interface, preprocessed to extract the number of reads assigned to each KEGG function (level 3; reads assigned to multiple annotations were discarded), and then transformed to percentage as explained previosly.

#### Real-world case study

Samples were processed with YAMP using the default parameters, as defined in the published YAMP configuration file, and the databases queried during the YAMP execution were deposited on Zenodo [63]. The Bray-Curtis dissimilarity values were evaluated using the species relative abundances as estimated by YAMP using MetaPhlAn2 [40] and the *vegdist* function in the vegan R package (version 2.4.3) [64]. PCoA was evaluated on the Bray-Curtis dissimilarity values using the *pcoa* function in the ape R package (version 4.1) [65]. Hierarchical clustering was computed using the Manhattan distance between species relative abundances and the *pvclust* function in the pvclust R package (version 2.0) [66].  A total of 10,000 bootstrap interactions were used to evaluate the *P* values supporting each cluster.

## Supplementary Material

GIGA-D-18-00004_Original_Submission.pdf

GIGA-D-18-00004_Revision_1.pdf

GIGA-D-18-00004_Revision_2.pdf

Response_to_Reviewer_Comments_Original_Submission.pdf

Response_to_Reviewer_Comments_Revision_1.pdf

Reviewer_1_Report_(Original_Submission) -- Konstantinos Krampis, PHD 01/28/2018 Reviewed

Reviewer_1_Report_(Revision_1) -- Konstantinos Krampis, PHD 12/05/2018 Reviewed

Reviewer_2_Report_(Original_Submission) -- Theodore Koutsandreas 02/01/2018 Reviewed

Supplement Files

## Availability of source code

- Project name: YAMP
- Project home page: https://github.com/alesssia/YAMP
- Operating system(s): UNIX-like systems, support for Amazon AWS Cloud
- Programming language: Bash, Nextflow
- Other requirements: Java, Docker/Singularity
- License: GNU GPL v3
- Any restrictions to use by non-academics: None
- Documentation and tutorials: https://github.com/alesssia/YAMP/wiki
- Scicrunch.org RRID:SCR_016236

## Availability of supporting data

Snapshots of the source code are available from the *GigaScience* GigaDB repository [67]. The Docker/Singularity image can be downloaded and installed from DockerHub [68]. The databases queried by YAMP during the simulation and real-world case studies are available from Zenodo [63].

## Abbreviations

GO: Gene Ontology; HMP: Human Microbiome Project; HPC: high-performance computing; KEEG: Kyoto Encyclopedia of Genes and Genomes; PCoA: principal coordinate analysis; PCR: polymerase chain reaction; QC: quality control; RMSE: root-mean-square error.

## References

[1] Baker M 1,500 scientists lift the lid on reproducibility. Nature News. 2016;533(7604):452. 10.1038/533452a 27225100

[2] Ioannidis JP, Allison DB, Ball CA, Repeatability of published microarray gene expression analyses. Nat Genet. 2009;41(2):149–55. 19174838 10.1038/ng.295

[3] Hothorn T, Leisch F Case studies in reproducibility. Brief Bioinform. 2011;12(3):288–300. 21278369 10.1093/bib/bbq084

[4] Peng RD Reproducible research in computational science. Science. 2011;334(6060):1226–7. 22144613 10.1126/science.1213847 PMC3383002

[5] Gronenschild EH, Habets P, Jacobs HI, The effects of FreeSurfer version, workstation type, and Macintosh operating system version on anatomical volume and cortical thickness measurements. PLoS One. 2012;7(6):e38234. 22675527 10.1371/journal.pone.0038234 PMC3365894

[6] Di Tommaso P, Chatzou M, Floden EW, Nextflow enables reproducible computational workflows. Nat Biotechnol. 2017;35(4):316–9. 28398311 10.1038/nbt.3820

[7] Leipzig J A review of bioinformatic pipeline frameworks. Brief Bioinform. 2017;18(3):530–6. 27013646 10.1093/bib/bbw020 PMC5429012

[8] Boettiger C An introduction to Docker for reproducible research. ACM SIGOPS Operating Sys Rev. 2015;49(1):71–9.

[9] Piccolo SR, Frampton MB Tools and techniques for computational reproducibility. GigaScience. 2016;5(1):30. 27401684 10.1186/s13742-016-0135-4 PMC4940747

[10] Davidson SB, Freire J Provenance and scientific workflows: challenges and opportunities. In: Proceedings of the 2008 ACM SIGMOD International Conference on Management of Data ACM; 2008 pp. 1345–50.

[11] Spjuth O, Bongcam-Rudloff E, Hernández GC, Experiences with workflows for automating data-intensive bioinformatics. Biol Direct. 2015;10(1):43. 26282399 10.1186/s13062-015-0071-8 PMC4539931

[12] Treangen TJ, Koren S, Sommer DD MetAMOS: a modular and open source metagenomic assembly and analysis pipeline. Genome Biol. 2013;14(1):R2. 23320958 10.1186/gb-2013-14-1-r2 PMC4053804

[13] Kultima JR, Coelho LP, Forslund K MOCAT2: a metagenomic assembly, annotation and profiling framework. Bioinformatics. 2016;32(16):2520–23. 27153620 10.1093/bioinformatics/btw183 PMC4978931

[14] Li W Analysis and comparison of very large metagenomes with fast clustering and functional annotation. BMC Biology. 2009;10(1):359. 10.1186/1471-2105-10-359 PMC2774329 19863816

[15] Markowitz VM, Chen IMA, Chu K, IMG/M 4 version of the integrated metagenome comparative analysis system. Nucleic Acids Res. 2013;42(D1):D568–73. 24136997 10.1093/nar/gkt919 PMC3964948

[16] Mitchell AL, , Scheremetjew M, Denise H EBI metagenomics in 2017: enriching the analysis of microbial communities, from sequence reads to assemblies. Nucleic Acids Res. 2017, D726–D735.;. 10.1093/nar/gkx967 PMC5753268 29069476

[17] Meyer F, Paarmann D, D’Souza M The metagenomics RAST server–a public resource for the automatic phylogenetic and functional analysis of metagenomes. BMC Biology. 2008;9(1):386. 10.1186/1471-2105-9-386 PMC2563014 18803844

[18] Wilke A, , Gerlach W, Harrison T, MG-RAST manual for version 4, revision 3; 2017. ftp://ftp.metagenomics.anl.gov/data/manual/mg-rast-tech-report-v4-r3.pdf, Accessed 21st June 2018

[19] Pérez-Wohlfeil E, Arjona-Medina JA, Torreno O Computational workflow for the fine-grained analysis of metagenomic samples. BMC Genomics. 2016;17(8):802. 27801291 10.1186/s12864-016-3063-x PMC5088524

[20] Angiuoli SV, Matalka M, Gussman A, CloVR: a virtual machine for automated and portable sequence analysis from the desktop using cloud computing. BMC Biology. 2011;12(1):356. 10.1186/1471-2105-12-356 PMC3228541 21878105

[21] Afgan E, Baker D, Van den Beek M The Galaxy platform for accessible, reproducible and collaborative biomedical analyses: 2016 update. Nucleic Acids Res. 2016;44(W1):W3–W10. 27137889 10.1093/nar/gkw343 PMC4987906

[22] Pond SK, Wadhawan S, Chiaromonte F Windshield splatter analysis with the Galaxy metagenomic pipeline. Genome Res. 2009;19(11):2144–53. 19819906 10.1101/gr.094508.109 PMC2775585

[23] White JR, Arze C, Matalka M CloVR-Metagenomics: functional and taxonomic microbial community characterization from metagenomic whole-genome shotgun (WGS) sequences–standard operating procedure, version 1.0. Nature Precedings. 2011.

[24] Ladoukakis E, Kolisis FN, Chatziioannou AA Integrative workflows for metagenomic analysis. Frontiers in Cell and Dev Bio. 2014;2, 70. 10.3389/fcell.2014.00070 PMC4237130 25478562

[25] Cohen-Boulakia S, Belhajjame K, Collin O Scientific workflows for computational reproducibility in the life sciences: status, challenges and opportunities. Future Generation Computer Systems. 2017;75:284–298.

[26] Ames SK, Gardner SN, Marti JM Using populations of human and microbial genomes for organism detection in metagenomes. Genome Res. 2015;25(7):1056–67. 25926546 10.1101/gr.184879.114 PMC4484388

[27] Blekhman R, Goodrich JK, Huang K, Host genetic variation impacts microbiome composition across human body sites. Genome Biol. 2015;16(1):191. 26374288 10.1186/s13059-015-0759-1 PMC4570153

[28] Xu H, Luo X, Qian J, FastUniq: a fast de novo duplicates removal tool for paired short reads. PLoS One. 2012;7(12):e52249. 23284954 10.1371/journal.pone.0052249 PMC3527383

[29] Jones MB, Highlander SK, Anderson EL Library preparation methodology can influence genomic and functional predictions in human microbiome research. Proc Nat Acad Sci. 2015;112(45):14024–9. 26512100 10.1073/pnas.1519288112 PMC4653211

[30] Docker. https://www.docker.com/, Accessed 21st June 2018

[31] Kurtzer GM, Sochat V, Bauer MW Singularity: scientific containers for mobility of compute. PLoS One. 2017;12(5):e0177459. 28494014 10.1371/journal.pone.0177459 PMC5426675

[32] Guzman C, D’Orso I CIPHER: a flexible and extensive workflow platform for integrative next-generation sequencing data analysis and genomic regulatory element prediction. BMC Biology. 2017;18(1):363. 10.1186/s12859-017-1770-1 PMC5549294 28789639

[33] Cario CL, Witte JS Orchid: a novel management, annotation, and machine learning framework for analyzing cancer mutations. Bioinformatics. 2018;34(6):936–942. 29106441 10.1093/bioinformatics/btx709 PMC5860353

[34] Sanderson ND, Street TL, Foster D Real-time analysis of nanopore-based metagenomic sequencing from orthopaedic device infection. bioRxiv. 2017; 220616. 10.1186/s12864-018-5094-y PMC6161345 30261842

[35] BitBucket Code Repository. https://bitbucket.org/, Accessed 21st June 2018.

[36] GitHub Code Repository. https://github.com/, Accessed 21st June 2018.

[37] GitLab Code Repository. https://about.gitlab.com/, Accessed 21st June 2018.

[38] Bushnell B BBMap short-read aligner, and other bioinformatics tools. 2015 https://sourceforge.net/projects/bbmap/, Accessed 21st June 2018.

[39] Andrews S FastQC a quality control tool for high throughput sequence data. 2010; http://www.bioinformatics.babraham.ac.uk/projects/fastqc/, Accessed 21st June 2018.

[40] Truong DT, Franzosa EA, Tickle TL MetaPhlAn2 for enhanced metagenomic taxonomic profiling. Nature Methods. 2015;12(10):902. 26418763 10.1038/nmeth.3589

[41] Consortium HMP Structure, function and diversity of the healthy human microbiome. Nature. 2012;486(7402):207–14. 22699609 10.1038/nature11234 PMC3564958

[42] Sczyrba A, Hofmann P, Belmann P, Critical assessment of metagenome interpretation–a benchmark of computational metagenomics software. Biorxiv. 2017; 099127. 10.1038/nmeth.4458 PMC5903868 28967888

[43] Abubucker S, Segata N, Goll J, HUMAnN2: the HMP Unified Metabolic Analysis Network 2; 2017 http://huttenhower.sph.harvard.edu/humann2.

[44] Abubucker S, Segata N, Goll J, Metabolic reconstruction for metagenomic data and its application to the human microbiome. PLos Comput Biol. 2012;8(6):e1002358. 22719234 10.1371/journal.pcbi.1002358 PMC3374609

[45] Caspi R, Altman T, Dreher K, The MetaCyc database of metabolic pathways and enzymes and the BioCyc collection of pathway/genome databases. Nucleic Acids Res. 2011;40(D1):D742–53. 22102576 10.1093/nar/gkr1014 PMC3245006

[46] Caporaso JG, Kuczynski J, Stombaugh J QIIME allows analysis of high-throughput community sequencing data. Nature Methods. 2010;7(5):335–6. 20383131 10.1038/nmeth.f.303 PMC3156573

[47] Schirmer M, Smeekens SP, Vlamakis H, Linking the human gut microbiome to inflammatory cytokine production capacity. Cell. 2016;167(4):1125–36. 27814509 10.1016/j.cell.2016.10.020 PMC5131922

[48] Piening BD, Zhou W, Contrepois K, Integrative personal omics profiles during periods of weight gain and loss. Cell Systems. 2018, 6, 2, 157–170.e8.;. 29361466 10.1016/j.cels.2017.12.013 PMC6021558

[49] Schulfer AF, Battaglia T, Alvarez Y, Intergenerational transfer of antibiotic-perturbed microbiota enhances colitis in susceptible mice. Nat Microbiol. 2018;3(2):234. 29180726 10.1038/s41564-017-0075-5 PMC5780248

[50] Bushnell B Introducing RemoveHuman: Human Contaminant Removal; 2014 http://seqanswers.com/forums/showthread.php?t=42552, Accessed 21st June 2018

[51] Zhou Q, Su X, Ning K Assessment of quality control approaches for metagenomic data analysis. Scientific Reports. 2014;4:6957. 25376098 10.1038/srep06957 PMC4223665

[52] Schmieder R, Edwards R Fast identification and removal of sequence contamination from genomic and metagenomic datasets. PLoS One. 2011;6(3):e17288. 21408061 10.1371/journal.pone.0017288 PMC3052304

[53] Human Microbiome Project Consortium. A framework for human microbiome research. Nature. 2012;486(7402):215. 22699610 10.1038/nature11209 PMC3377744

[54] Khazina E, Weichenrieder O Non-LTR retrotransposons encode noncanonical RRM domains in their first open reading frame. Proc Nat Acad Sci. 2009;106(3):731–6. 19139409 10.1073/pnas.0809964106 PMC2630067

[55] Gomez-Alvarez V, Teal TK, Schmidt TM Systematic artifacts in metagenomes from complex microbial communities. ISME Journal. 2009;3(11):1314. 19587772 10.1038/ismej.2009.72

[56] Niu B, Fu L, Sun S Artificial and natural duplicates in pyrosequencing reads of metagenomic data. BMC Biology. 2010;11(1):187. 10.1186/1471-2105-11-187 PMC2874554 20388221

[57] Overbeek R, Begley T, Butler RM The subsystems approach to genome annotation and its use in the project to annotate 1000 genomes. Nucleic Acids Res. 2005;33(17):5691–5702. 16214803 10.1093/nar/gki866 PMC1251668

[58] Aagaard K, Ma J, Antony KM, The placenta harbors a unique microbiome. Science Translational Medicine. 2014;6(237):237ra65–237ra65. 10.1126/scitranslmed.3008599 PMC4929217 24848255

[59] Freedman LP, Cockburn IM, Simcoe TS The economics of reproducibility in preclinical research. PLoS Biology. 2015;13(6):e1002165. 26057340 10.1371/journal.pbio.1002165 PMC4461318

[60] Garijo D, Kinnings S, Xie L, Quantifying reproducibility in computational biology: the case of the tuberculosis drugome. PLoS One. 2013;8(11):e80278. 24312207 10.1371/journal.pone.0080278 PMC3842296

[61] Narayanasamy S, Jarosz Y, Muller EE IMP: a pipeline for reproducible reference-independent integrated metagenomic and metatranscriptomic analyses. Genome Biol. 2016;17(1):260. 27986083 10.1186/s13059-016-1116-8 PMC5159968

[62] Kim B, Ali T, Lijeron C Bio-Docklets: virtualization containers for single-step execution of NGS pipelines. GigaScience. 2017;6(8):1–7. 10.1093/gigascience/gix048 PMC5569920 28854616

[63] Visconti A Data for YAMP, Zenodo; 2017 https://zenodo.org/record/1068229, Accessed 21st June 2018.

[64] Dixon P VEGAN, a package of R functions for community ecology. Journal Vegetation Science. 2003;14(6):927–30.

[65] Paradis E, Claude J, Strimmer K APE: analyses of phylogenetics and evolution in R language. Bioinformatics. 2004;20(2):289–90. 14734327 10.1093/bioinformatics/btg412

[66] Suzuki R, Shimodaira H Pvclust: an R package for assessing the uncertainty in hierarchical clustering. Bioinformatics. 2006;22(12):1540–2. 16595560 10.1093/bioinformatics/btl117

[67] Visconti A, Martin TC, Falchi M, Supporting data for “YAMP: a containerized workflow enabling reproducibility in metagenomics research.”. GigaScience Database. 2018 http://dx.doi.org/10.5524/100459. 10.1093/gigascience/giy072 PMC6047416 29917068

[68] Visconti A YAMP Docker image; DockerHub; 2017 https://hub.docker.com/r/alesssia/yampdocker, Accessed 21st June 2018

