# Supplementary material for: YAMP: a containerized workflow enabling reproducibility in metagenomics research
Source: Gigascience. 2018 Jun 18;7(7):giy072. doi: 10.1093/gigascience/giy072 (PMC6047416; doi:10.1093/gigascience/giy072)
Supplement: GIGA-D-18-00004_Original_Submission.pdf [file giy072_giga-d-18-00004_original_submission.pdf]

GigaScience

YAMP: a containerised work flow enabling reproducibility in metagenomics research

--Manuscript Draft--

**Manuscript Number:**	GIGA-D-18-00004
**Full Title:**	YAMP: a containerised work flow enabling reproducibility in metagenomics research
**Article Type:**	Technical Note
**Funding Information:**	Medical Research Council (MR/M004422/1) — Not applicable
**Abstract:**	YAMP is a user-friendly workflow that enables the analysis of whole shotgun metagenomics data while using containerisation to ensure computational reproducibility and facilitate collaborative research. YAMP can be executed on any UNIX-like system, and offers seamless support for multiple job schedulers as well as for Amazon AWS cloud. Although YAMP has been developed to be ready-to-use by non-experts, bioinformaticians will appreciate its flexibility, modularisation, and simple customisation.<br><br>The YAMP script, parameters, and documentation are available at https://github.com/alesssia/YAMP.
**Corresponding Author:**	Alessia Visconti<br><br>UNITED KINGDOM
**Corresponding Author Secondary Information:**	
**Corresponding Author's Institution:**	
**Corresponding Author's Secondary Institution:**	
**First Author:**	Alessia Visconti
**First Author Secondary Information:**	
**Order of Authors:**	Alessia Visconti
	Tiphaine C Martin
	Mario Falchi
**Order of Authors Secondary Information:**	
**Opposed Reviewers:**	
**Additional Information:**	
**Question**	**Response**
Are you submitting this manuscript to a special series or article collection?	No
**Experimental design and statistics**<br><br>Full details of the experimental design and statistical methods used should be given in the Methods section, as detailed in our Minimum Standards Reporting Checklist. Information essential to interpreting the data presented should be made available in the figure legends.<br><br>Have you included all the information	Yes

Powered by Editorial Manager® and ProduXion Manager® from Aries Systems Corporation

requested in your manuscript?	
**Resources**<br><br>A description of all resources used, including antibodies, cell lines, animals and software tools, with enough information to allow them to be uniquely identified, should be included in the Methods section. Authors are strongly encouraged to cite Research Resource Identifiers (RRIDs) for antibodies, model organisms and tools, where possible.<br><br>Have you included the information requested as detailed in our Minimum Standards Reporting Checklist?	Yes
**Availability of data and materials**<br><br>All datasets and code on which the conclusions of the paper rely must be either included in your submission or deposited in publicly available repositories (where available and ethically appropriate), referencing such data using a unique identifier in the references and in the “Availability of Data and Materials” section of your manuscript.<br><br>Have you have met the above requirement as detailed in our Minimum Standards Reporting Checklist?	Yes

Manuscript

Click here to download Manuscript main.pdf

GigaScience, 2017, 1–7

doi: xx.xxxx/xxxx
Manuscript in Preparation
Technical note

TECHNICAL NOTE

YAMP: a containerised workflow enabling reproducibility in metagenomics research

Alessia Visconti[1,*], Tiphaine C. Martin[1] and Mario Falchi[1]

[1]Department of Twin Research and Genetic Epidemiology, King's College London, London, UK

[*]alessia.visconti@kcl.ac.uk

Abstract

YAMP is a user-friendly workflow that enables the analysis of whole shotgun metagenomics data while using containerisation to ensure computational reproducibility and facilitate collaborative research. YAMP can be executed on any UNIX-like system, and offers seamless support for multiple job schedulers as well as for Amazon AWS cloud. Although YAMP has been developed to be ready-to-use by non-experts, bioinformaticians will appreciate its flexibility, modularisation, and simple customisation.
The YAMP script, parameters, and documentation are available at `https://github.com/alesssia/YAMP`.

Key words: Metagenomics; Reproducibility; Workflow; Containerisation; Docker

Findings

Background

Thanks to the increased cost-effectiveness of high-throughput technologies, the number of studies collecting and analysing large amounts of data has surged, opening new challenges for data analysis and research reproducibility. A ubiquitous lack of repeatability and reproducibility has in fact been observed, and a recent Nature's survey of 1,576 researchers showed that more than 50% and 70% of them failed to reproduce their own and other scientists' experiments, respectively [1]. Unavailability of primary data and computational experimentation have been named as the major culprits for this reproducibility crisis, with many studies relying on *ad hoc* scripts and not publishing the necessary code and/nor sufficient details to reproduce the reported results [2, 3, 4], and with variations across workstations and operating systems representing another obstacle [5, 6]. To overcome this issue, tools allowing the development of workflows [7] and software containers [8] have been proposed [9]. In fact, containerised well-structured workflows allow storing every detail of the workflow execution, including software's versions and parameters (*provenance*, [10]), and nullify systems' variations [6], guaranteeing studies' repeatability and reproducibility. Containerised workflows also facilitate collaborative projects, by ensuring identical analysis processes, thus comparable results, and allow the automatisation of data-intensive repetitive tasks [11]. Moreover, they save users with little bioinformatics or computational expertise from the hassles of installing the required pieces of software, and of designing and implementing often complex analysis orchestrations, while expert bioinformaticians can use them as a starting point for customised analyses, thus avoiding redundant solutions.

In metagenomics research, several analysis pipelines have been developed so far. However, they either do not support containerisation (*e.g.*, MetAMOS [12], MOCAT2 [13], RAMMCAP [14]), thus potentially compromising reproducibility, or require users to upload their unpublished and/or confidential data on third-party servers (*e.g.*, IMG/M [15], the EBI metagenomic pipeline [16], and MG-RAST [17]), where, according to the available resources, they can spend several days waiting to be processed [18], and with data privacy concerning some of the researchers [19]. Scalable metagenomics pipelines allowing both local and cloud execution have been proposed, such as CloVR-Metagenomics [20], and those implemented using the Galaxy platform [21, 22]. However, the former lacks steps for quality control (QC) and functional characterisation, and the latter requires non-trivial expertise for local installation [23], with porting issues observed among different Galaxy versions [24]. QC is also often overlooked. For instance, both

Compiled on: December 14, 2017.
Draft manuscript prepared by the author.

MetAMOS and the EBI metagenomic pipeline do not include a step for removing contaminant genomes, with the latter also not discarding identical duplicates. Ignoring decontamination may lead to reads not belonging to the studied ecosystem to be used in downstream analyses, causing potential mismapping on reference databases and, thus, erroneous functional profiling. Retaining duplicated reads, usually considered as technical artefacts derived from PCR amplification [25], may hamper the correct estimation of both community composition and functional capabilities. Finally, MG-RAST performs de-duplication after quality trimming, potentially introducing biases due to the fact that trimming, by modifying the read sequence, may mask true duplicates or generate false ones.

Here we present *"Yet Another Metagenomic Pipeline"* (YAMP), a ready-to-use containerised workflow that, using state-of-the-art tools, processes raw shotgun metagenomics sequencing data up to the taxonomic and functional annotation. YAMP is implemented in Nextflow [6] and it is accompanied by a Docker (https://www.docker.com) and a Singularity (http://singularity.lbl.gov, [26]) container. While the former defines a platform-independent virtualised light-weight operating system that includes all the pieces of software required by YAMP and traces their versioning, the latter allows these features to be transferred to High Performance Computing (HPC) systems, with which Docker is inherently incompatible.

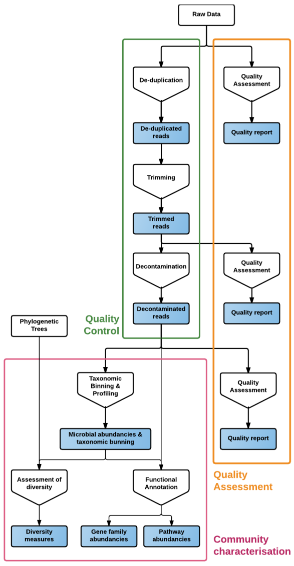

Figure 1. The YAMP workflow. White rectangles represent data to be provided as input, and blue rectangles those produced in output. Pentagons represent the analysis steps.

The YAMP workflow

The YAMP workflow is composed of three analysis blocks: the quality control, (Figure 1, green rectangle), complemented by several steps of assessment and visualisation of data quality (Figure 1, orange rectangle), and the community characterisation (Figure 1, pink rectangle).

The QC starts with an optional step of de-duplication, where identical reads, potentially generated by PCR amplification, are removed. The optionality of this step allows retaining *natural duplicates* when PCR-free library preparation approaches (*e.g.*, TruSeq) are used. Next, reads are first filtered to remove adapters, known artefacts, phiX, and then quality-trimmed. Notably, YAMP removes duplicates before trimming, avoiding the introduction of biases due to the reads' sequence modifications. Reads that become too short after trimming are discarded. Indeed, they may map to multiple genomes or genomic regions and compromise downstream analyses. When paired-end reads are at hand, singleton reads (*i.e.*, paired-end reads whose mates have been removed) are preserved in order to retain as much information as possible. Finally, reads are screened for contaminants, *e.g.*, reads that do not belong to the studied ecosystem. The implemented QC is accompanied by multiple steps which assess and visualise the reads' quality, in order to evaluate the quality of the raw data and effectiveness of the trimming and decontamination step. The QC is followed by multiple steps aimed at estimating multiple α-diversity measures, and at characterising the taxonomic and functional profiles of the microbial community, *i.e.* at identifying and quantifying the micro-organisms present in the metagenomic sample (taxonomic binning and profiling) and their functional capabilities (functional characterisation).

Implementation

YAMP is developed in Nextflow, a workflow management system that allows the effortlessly developing, deploying, and executing of complex distributed computational workflows [6], and which has been used in several life-science projects (*e.g.*, [27, 28, 29]). Nextflow allows for user-transparent high-level parallelisation, and offers out-of-the-box support for distributed computational environments, ensuring the scalability of large projects. Its executor allows porting of workflows on any UNIX-based system (*e.g.*, local machine, HPC facilities) in a seamless fashion. Reproducibility is guaranteed by a user-transparent integration with Docker/Singularity and with the BitBucket (https://bitbucket.org/), GitHub (https://github.com/), and GitLab (https://about.gitlab.com/) code repositories, therefore ensuring a consistent tracking of both software and code version. The so-called *retrospective provenance*, *i.e.*, the description of each completed analysis step along with details about its underlying execution environment [10], is captured by task execution reports, which record, among the others, the exact command executed, the tasks' working directory, environment and output, as well as the container's image.

YAMP integrates state-of-the-art tools for the analysis of metagenomics data. The QC is performed by means of a number of tools belonging to the BBmap suite [30], namely clumpify, BBduk, and BBwrap, which are well-established, and allow processing both single- and paired-end reads from all the major sequencing platforms (*i.e.*, Illumina, Roche 454 pyrosequencing, Sanger, Ion Torrent, PacBio, and Oxford Nanopore). They are also computationally efficient, thus scalable to large metagenomics projects and samples. FastQC [31], which provides very detailed reports on reads' quality, is used to perform QC assessment and visualisation. Taxonomic binning and profiling is performed with MetaPhlAn2 [32], which uses clade-specific markers to both detect the microorganisms and to estimate their relative abundance. The clade-based approach implemented in MetaPhlAn2 has been shown to scale to large datasets, and to be effective to quantitatively profile the microbial composition during the Human Microbiome Project (HMP) [33] and the Critical Assessment of Metagenome Interpretation (CAMI) challenge [34]. Moreover, MetaPhlAn2 is becoming the *de facto* standard when samples belong to well-characterised environments [35]. The functional capabilities of the microbial community are assessed by the HUMAnN2 pipeline [36], an extension of the pipeline originally developed by the HMP Metabolic Reconstruction Working Group to infer the functional and metabolic potential of microbial communities during the HMP [37]. Briefly, the HUMAnN2 pipeline first stratifies the community in known and unclassified organisms using the MetaPhlAn2 results and the

```
---------------------------------------------
YET ANOTHER METAGENOMIC PIPELINE (YAMP) - Version: 0.9.4 (20171207)
---------------------------------------------

[... wrapped text ...]

++++++++++++++++++++++++++++++++++++++++++++++++++++++++++++++++++++++++++++

Analysis starting at Thu Dec 14 16:10:45 GMT 2017 by user: alessia
Analysed sample(s): /Users/alessia/yamp/data/SRR1944683_1.fastq.gz and
/Users/alessia/yamp/data/SRR1944683_2.fastq.gz
Results will be saved at /Users/alessia/yamp/data/Anterior_nares_2
New files will be saved using the 'Anterior_nares_2' prefix

Analysis mode? complete
Library layout? paired
Saving QC temporary files? true
Saving community characterisation temporary files? true
Performing de-duplication? true

------------

Analysis introspection:

Operating System:
    name:         Linux
    architecture: amd64
    version:      2.6.32-431.17.1.el6.x86_64

Java
    version: 1.8.0_65
    Java Virtual Machine: Java HotSpot(TM) 64-Bit Server VM ; version: 25.65-b01

nextflow:
    version:   0.25.4
    build:     4512
    timestamp: 31-07-2017 10:25 UTC

[... wrapped text ...]

Analysis environment:

    projectDir: /Users/alessia/yamp
    launchDir:  /Users/alessia/yamp/data
    workingDir: /Users/alessia/yamp/data/tmp/Anterior_nares_2_work

    command line: nextflow run /Users/alessia/yamp/pipeline.nf --reads1 /Users/alessia/yamp/data/SRR1944683_1.fastq.gz --reads2
    /Users/alessia/yamp/data/SRR1944683_2.fastq.gz --prefix Anterior_nares_2 --outdir /Users/alessia/yamp/data  --mode complete

    Run name:   pensive_montalcini
    Session ID: 9929ee81-a95b-40ed-99cb-9a54d1506852
    profile:    standard

++++++++++++++++++++++++++++++++++++++++++++++++++++++++++++++++++++++++++++

Performing Quality Control. [Assessment of read quality] at Thu Dec 14 16:10:45 GMT 2017
File being analysed: SRR1944683_1.fastq.gz

Using FastQC v0.11.3
Executing command fastqc --quiet --noextract --format fastq --outdir=. --threads 1 SRR1944683_1.fastq.gz

[... wrapped text ...]

++++++++++++++++++++++++++++++++++++++++++++++++++++++++++++++++++++++++++++

Performing Quality Control. STEP 1 [De-duplication] at Thu Dec 14 16:10:45 GMT 2017

Using clumpify.sh in BBMap version 37.10
Executing command: clumpify.sh -Xmx32G in1=SRR1944683_1.fastq.gz in2=SRR1944683_2.fastq.gz out1=Anterior_nares_2_dedupe_R1.fq.gz
out2=Anterior_nares_2_dedupe_R2.fq.gz qin=33 dedupe subs=0 threads=4

Clumpify's de-duplication stats:

Reads In:            5641800
Clumps Formed:         85885
Duplicates Found:    5439512

202288 out of 5641800 paired reads survived de-duplication (3.58552%, 5439512 reads removed)

STEP 1 (Quality control) terminated at Thu Dec 14 16:11:04 GMT 2017 (18.931907299 seconds)
```

Figure 2. Example of an excerpt of the YAMP execution log.

ChocoPhlAn pan-genome database, and then combines these results with those obtained through an organism-agnostic search on the UniRef proteomic database. The identified taxonomic profile is additionally used by YAMP to evaluate multiple α-diversity measures through functions available within the widely-used QIIME pipeline [38], which has been designed to analyse amplicon (*e.g.*, 16S or 18S rRNA genes) sequencing data.

YAMP Input/Output

YAMP accepts in input both single- and paired-end FASTQ files, and users can customise the workflow execution either by using command line options or by modifying a simple plain-text configuration file, where parameters are set as key-value pairs. While the parameters could be tuned according to the dataset at hand, to facilitate non-expert users in their analyses we provide a set of default parameters derived from our own analysis experience. The output generated by YAMP includes a FASTQ file of QC'ed reads, the taxonomy composition along with the microbe, gene and pathway relative abundances, the pathway coverage, and multiple α-diversity measures. An option allows users to retain temporary files, such as those generated by the QC steps or during the HUMAnN2 execution. Additionally, YAMP outputs several QC reports, a very detailed log file recording information about each analysis step which ensures the retrospective provenance (Figure 2), and statistics of memory usage and time of execution (Figure 3).

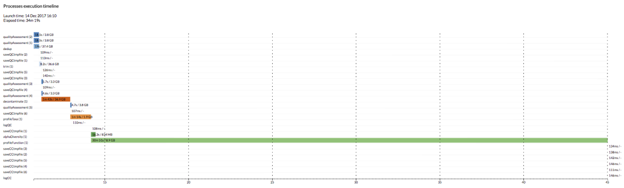

Figure 3. Example of YAMP execution profile. YAMP returns the time spend during its complete execution and in each step, as well as the steps' memory peaks.

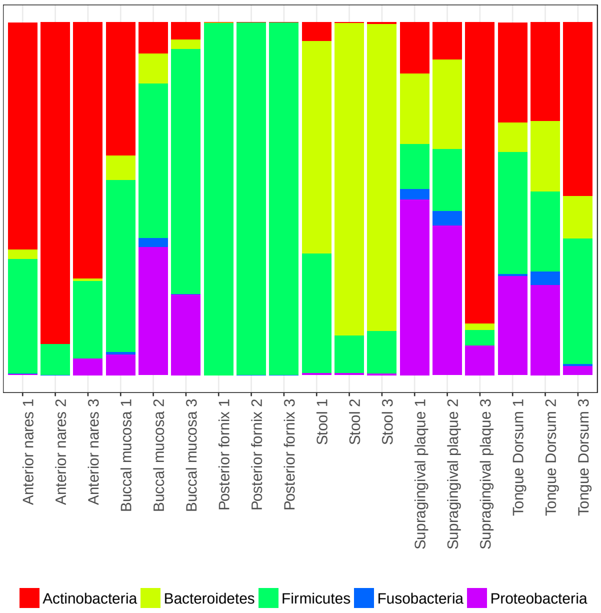

Figure 4. Phylum level relative abundances. Each vertical bar represents a sample. Phylum relative abundances were estimated by YAMP using MetaPhlAn2. Unspecified viral phyla are not shown.

Results

YAMP is based on well-established metagenomics tools and here, to facilitate the discussion on YAMP computational requirements, and to assess its ability to reproduce research results already described in the literature, we analysed 18 randomly selected samples from six different body sites sequenced during the Phase III of the Human Microbiome Project [39] (Table 1). On average, the selected samples included 12.6M paired-end reads (25.2M reads in total), which yielded to 13.3M QC'ed reads (including both paired-end and singleton reads), and were processed in an average time of two hours using four threads on a machine sporting a 2.60GHz Intel® Xeon® processor with 32 GB of RAM (Table 1). At the phylum level, each body site showed a characteristic signature (Figure 4), with a predominance of Actinobacteria in the airways, Firmicutes in the vagina, Bacteroidetes in the stool, and a mixture of Actinobacteria, Firmicutes and Proteobacteria in the oral cavity, as already observed in previous studies [40]. A site-specific microbial signature was also present at the species level, where both the Principal coordinate analysis (PCoA) evaluated using the Bray-Curtis dissimilarity (Supplementary Figure S1 and S2), and the hierarchical clustering computed on the Manhattan distances between species relative abundances (Supplementary Figure S3) showed that the taxonomy composition was sufficient to discriminate among body sites, even though it had limited ability in distinguishing among different loci in the oral cavity.

Discussion

In conclusion, with YAMP, we provide a user-friendly workflow that enables the analysis of whole shotgun metagenomics data. By supporting containerisation, YAMP allows for computational reproducibility, also enabling collaborative studies. In fact, while software versions are described in the Docker/Singularity container, the Nextflow script and configuration file capture all the details needed to fully track each step of data processing, thus satisfying the prospective provenance requirements, while the very detailed YAMP log file ensures retrospective provenance. Indeed, to ensure reproducibility, researchers should only provide the YAMP configuration file and a link to the container image. Being based on Nextflow, YAMP runs on any UNIX-like system, provides out-of-the-box support for several job schedulers (*e.g.,* PBS, SGE, SLURM) and for Amazon AWS cloud, and its integration with Docker/Singularity is completely user-transparent. Finally, while YAMP has been developed to be ready to use by non-experts, and potentially does not require any software installation or parameter tuning, bioinformaticians will value its flexibility and simple customisation. In fact, the well-defined YAMP modularisation and the usage of standard data formats allow both an easy integration of new analysis steps and a customisation of the existing ones. This is of particular importance in a fast-developing field such as metagenomics, where the analysis guidelines and tools and are not stable yet. With YAMP we provide a workflow to which new analysis modules can be easily added, and where tools that become outdated can be effortlessly replaced, therefore securing its sustainability.

YAMP is made available as a Nextflow script that allows a user-friendly execution via the command line. The source code is available in the YAMP GitHub repository (https://github.com/alesssia/YAMP), which includes a wiki with a full documentation and several tutorials. The Docker/Singularity image can be downloaded and installed from DockerHub (https://hub.docker.com/r/alesssia/yampdocker).

Potential implications

YAMP has been designed with the specific goals of enabling reproducible metagenomics analyses, facilitating collaborative projects, and helping researchers with limited computational experience who are approaching this field of research. However, we are confident that other areas of research would be aided by a more widespread use of containerised well-structured workflows. Indeed, as outlined in the Background Section, a lack of reproducibility is nowadays ubiquitous, and, besides undermining the credibility of scientific research, it has an economical cost, quantified, for instance, in US$28B/year for preclinical research [41]. On the other hand, ensuring reproducibility does not come for free: anecdotic evidence suggests that the time spent on a project may increase by 30-50% [1], and that to reproduce the analysis of single computational biology paper can require up to 280 hours [42]. YAMP represents a proof-of-concept showing a simple way to enable reproducible and collaborative research. We also advocate the sharing of such containerised workflows, which will benefit a wide group of researchers, regardless of their computational experience [11].

Methods

Data Availability

The 18 randomly selected samples used to assess YAMP belong to the Phase III of the Human Microbiome Project [39], and were downloaded from the European Nucleotide Archive website (Study accession number: PRJNA275349, https://www.ebi.ac.uk/ena/data/view/PRJNA275349). Samples were collected from healthy adults residing in the USA at the time of sample collection. After genomic DNA extraction, metagenomics library preparation was performed using the NexteraXT library construction protocol. Paired-end metagenomics sequencing was performed on the Illumina HiSeq2000 platform with a read length of 100 bp. Samples' accession numbers are reported in Table 1.

Data Analysis

Samples were processed with YAMP using the default parameters, as defined in the published YAMP configuration file (https://raw.githubusercontent.com/alesssia/YAMP/master/nextflow.config). The Bray-Curtis dissimilarity values were evaluated using the species relative abundances as estimated by YAMP using MetaPhlAn2 [32] and the *vegdist* function in the vegan R package (version 2.4.3) [43]. Principal coordinate analysis (PCoA) was evaluated on the Bray-Curtis dissimilarity values using the *pcoa* function in the ape R package (version 4.1) [44]. Hierarchical clustering was computed using the Manhattan distance between species relative abundances and the *pvclust* function in the pvclust R package (version 2.0) [45]. 10,000 bootstrap interactions were used to evaluate the P values supporting each cluster.

Availability of source code and requirements

- Project name: YAMP
- Project home page: https://github.com/alesssia/YAMP
- Operating system(s): UNIX-like systems, support for Amazon AWS Cloud
- Programming language: Nextflow
- Other requirements: Docker/Singularity
- License: GNU GPL v3
- Any restrictions to use by non-academics: None

Declarations

List of abbreviations

HPC: High Performance Computing; PCoA: Principal coordinate analysis; QC: Quality Control.

Ethical Approval

Not applicable.

Consent for publication

Not applicable.

Competing Interests

The authors declare that they have no competing interests.

Author's Contributions

AV, TCM, and MF designed the metagenomic workflow. AV implemented and optimised the workflow, created the Docker container, and wrote the manuscript. All the authors read, commented, and approved the final manuscript.

Acknowledgements

AV thanks Paolo Di Tommaso and Brian Bushnell for their help with Nextflow and BBmap.

This work has been funded by the Medical Research Council, grant: MR/M004422/1. TwinsUK is funded by the Wellcome Trust, Medical Research Council, European Union, the National Institute for Health Research (NIHR)-funded BioResource, Clinical Research Facility and Biomedical Research Centre based at Guy's and St Thomas' NHS Foundation Trust in partnership with King's College London

References

1. Baker M. 1,500 scientists lift the lid on reproducibility. Nature News 2016;533(7604):452.
2. Ioannidis JP, Allison DB, Ball CA, Coulibaly I, Cui X, Culhane AC, et al. Repeatability of published microarray gene expression analyses. Nature genetics 2009;41(2):149–155.
3. Hothorn T, Leisch F. Case studies in reproducibility. Briefings in bioinformatics 2011;12(3):288–300.
4. Peng RD. Reproducible research in computational science. Science 2011;334(6060):1226–1227.
5. Gronenschild EH, Habets P, Jacobs HI, Mengelers R, Rozendaal N, Van Os J, et al. The effects of FreeSurfer version, workstation type, and Macintosh operating system version on anatomical volume and cortical thickness measurements. PloS one 2012;7(6):e38234.
6. Di Tommaso P, Chatzou M, Floden EW, Barja PP, Palumbo E, Notredame C. Nextflow enables reproducible computational workflows. Nature Biotechnology 2017;35(4):316–319.
7. Leipzig J. A review of bioinformatic pipeline frameworks. Briefings in bioinformatics 2017;18(3):530–536.
8. Boettiger C. An introduction to Docker for reproducible research. ACM SIGOPS Operating Systems Review 2015;49(1):71–79.
9. Piccolo SR, Frampton MB. Tools and techniques for computational reproducibility. GigaScience 2016;5(1):30.
10. Davidson SB, Freire J. Provenance and scientific workflows: challenges and opportunities. In: Proceedings of the 2008 ACM SIGMOD international conference on Management of data ACM; 2008. p. 1345–1350.
11. Spjuth O, Bongcam-Rudloff E, Hernández GC, Forer L, Giovacchini M, Guimera RV, et al. Experiences with workflows for automating data-intensive bioinformatics. Biology direct 2015;10(1):43.
12. Treangen TJ, Koren S, Sommer DD, Liu B, Astrovskaya I, Ondov B, et al. MetAMOS: a modular and open source metagenomic assembly and analysis pipeline. Genome biology 2013;14(1):R2.
13. Kultima JR, Coelho LP, Forslund K, Huerta-Cepas J, Li SS, Driessen M, et al. MOCAT2: a metagenomic assembly, annotation and profiling framework. Bioinformatics 2016;32(16):2520–2523.
14. Li W. Analysis and comparison of very large metagenomes with fast clustering and functional annotation. BMC bioinformatics 2009;10(1):359.
15. Markowitz VM, Chen IMA, Chu K, Szeto E, Palaniappan K, Pillay M, et al. IMG/M 4 version of the integrated metagenome comparative analysis system. Nucleic Acids Research 2013;42(D1):D568–D573.
16. Mitchell AL, Scheremetjew M, Denise H, Potter S, Tarkowska A, Qureshi M, et al. EBI Metagenomics in 2017: enriching the analysis of microbial communities, from sequence reads to assemblies. Nucleic acids research 2017;.
17. Meyer F, Paarmann D, D'Souza M, Olson R, Glass EM, Kubal M, et al. The metagenomics RAST server–a public resource for the automatic phylogenetic and functional analysis of metagenomes. BMC bioinformatics 2008;9(1):386.
18. Wilke A, Gerlach W, Harrison T, Paczian T, Trimble WL, Meyer F, MG-RAST Manual for version 4, revision 3; 2017. ftp://ftp.metagenomics.anl.gov/data/manual/mg-rast-manual.pdf.
19. Pérez-Wohlfeil E, Arjona-Medina JA, Torreno O, Ulzurrun E, Trelles O. Computational workflow for the fine-grained analysis of metagenomic samples. BMC genomics 2016;17(8):802.
20. Angiuoli SV, Matalka M, Gussman A, Galens K, Vangala M, Riley DR, et al. CloVR: a virtual machine for automated and portable sequence analysis from the desktop using cloud computing. BMC bioinformatics 2011;12(1):356.
21. Afgan E, Baker D, Van den Beek M, Blankenberg D, Bouvier D, Čech M, et al. The Galaxy platform for accessible, reproducible and collaborative biomedical analyses: 2016 update. Nucleic acids research 2016;44(W1):W3–W10.
22. Pond SK, Wadhawan S, Chiaromonte F, Ananda G, Chung WY, Taylor J, et al. Windshield splatter analysis with the Galaxy metagenomic pipeline. Genome research 2009;19(11):2144–2153.
23. Ladoukakis E, Kolisis FN, Chatziioannou AA. Integrative workflows for metagenomic analysis. Frontiers in cell and developmental biology 2014;2.
24. Cohen-Boulakia S, Belhajjame K, Collin O, Chopard J, Froidevaux C, Gaignard A, et al. Scientific workflows for computational reproducibility in the life sciences: Status, challenges and opportunities. Future Generation Computer Systems 2017;.
25. Xu H, Luo X, Qian J, Pang X, Song J, Qian G, et al. FastUniq: a fast de novo duplicates removal tool for paired short reads. PloS one 2012;7(12):e52249.
26. Kurtzer GM, Sochat V, Bauer MW. Singularity: Scientific containers for mobility of compute. PloS one 2017;12(5):e0177459.
27. Guzman C, D'Orso I. CIPHER: a flexible and extensive workflow platform for integrative next-generation sequencing data analysis and genomic regulatory element prediction. BMC bioinformatics 2017;18(1):363.
28. Cario CL, Witte JS. Orchid: a novel management, annotation, and machine learning framework for analyzing cancer mutations. Bioinformatics 2017;.
29. Sanderson ND, Street TL, Foster D, Swann J, Atkins BL, Brent AJ, et al. Real-time analysis of nanopore-based metagenomic sequencing from orthopaedic device infection. bioRxiv 2017;p. 220616.
30. Bushnell B, BBMap short-read aligner, and other bioinformatics tools; 2015. https://sourceforge.net/projects/bbmap/.
31. Andrews S, FastQC A Quality Control tool for High Throughput Sequence Data; 2010. http://www.bioinformatics.babraham.ac.uk/projects/fastqc/.
32. Truong DT, Franzosa EA, Tickle TL, Scholz M, Weingart G, Pasolli E, et al. MetaPhlAn2 for enhanced metagenomic taxonomic profiling. Nature methods 2015;12(10):902.
33. Consortium HMP, et al. Structure, function and diversity of the healthy human microbiome. Nature 2012;486(7402):207–214.

34. Sczyrba A, Hofmann P, Belmann P, Koslicki D, Janssen S, Droege J, et al. Critical Assessment of Metagenome Interpretation- a benchmark of computational metagenomics software. Biorxiv 2017;p. 099127.
35. Quince C, Walker AW, Simpson JT, Loman NJ, Segata N. Shotgun metagenomics, from sampling to analysis. Nature Biotechnology 2017;35(9):833–844.
36. Abubucker S, Segata N, Goll J, Schubert AM, Izard J, Cantarel BL, et al., HUMAnN2: The HMP Unified Metabolic Analysis Network 2; 2017. http://huttenhower.sph.harvard.edu/humann2.
37. Abubucker S, Segata N, Goll J, Schubert AM, Izard J, Cantarel BL, et al. Metabolic reconstruction for metagenomic data and its application to the human microbiome. PLoS computational biology 2012;8(6):e1002358.
38. Caporaso JG, Kuczynski J, Stombaugh J, Bittinger K, Bushman FD, Costello EK, et al. QIIME allows analysis of high-throughput community sequencing data. Nature methods 2010;7(5):335–336.
39. The Human Microbiome Project Consortium. A framework for human microbiome research. Nature 2012;486(7402):215.
40. Aagaard K, Ma J, Antony KM, Ganu R, Petrosino J, Versalovic J. The placenta harbors a unique microbiome. Science translational medicine 2014;6(237):237ra65–237ra65.
41. Freedman LP, Cockburn IM, Simcoe TS. The economics of reproducibility in preclinical research. PLoS biology 2015;13(6):e1002165.
42. Garijo D, Kinnings S, Xie L, Xie L, Zhang Y, Bourne PE, et al. Quantifying reproducibility in computational biology: the case of the tuberculosis drugome. PloS one 2013;8(11):e80278.
43. Dixon P. VEGAN, a package of R functions for community ecology. Journal of Vegetation Science 2003;14(6):927–930.
44. Paradis E, Claude J, Strimmer K. APE: analyses of phylogenetics and evolution in R language. Bioinformatics 2004;20(2):289–290.
45. Suzuki R, Shimodaira H. Pvclust: an R package for assessing the uncertainty in hierarchical clustering. Bioinformatics 2006;22(12):1540–1542.

Table 1. Run Accession Number and statistics for 18 randomly selected samples from the Human Microbiome Project (HMP) Phase III [39]. Samples were processed using 4 threads on a machine sporting a 2.60GHz Intel® Xeon® processor with 32 GB of RAM.

Body site	Locus	SRA Accession Number	Number of Raw Paired-end Reads	Number of QC'ed Reads Paired-ed ; Singletons	Running time
Airways	Anterior nares	SRR1944674	1,181,169	590,714 ; 42,241	39m 02s
		SRR1944683	2,820,900	56,151 ; 9,513	31m 31s
		SRR1952439	14,635,701	201,260 ; 17,345	42m 00s
Gut	Stool	SRR1951826	7,956,274	7,121,697 ; 494,289	2h 15m 39s
		SRR1944873	11,033,130	9,796,817 ; 942,566	2h 26m 01s
		SRR1952058	5,834,232	5,484,362 ; 248,819	1h 39m 10s
Oral cavity	Buccal mucosa	SRR1944703	6,231,553	285,906 ; 24,212	39m 09s
		SRR1952437	15,361,468	3,451,844 ; 149,714	1h 19m 26s
		SRR1952380	11,872,420	631,595 ; 41,957	49m 07s
	Supragingival plaque	SRR1952435	16,169,911	13,620,835 ; 672,610	2h 44m 56s
		SRR1952436	21,971,588	17,237,506 ; 987,950	4h 07m 11s
		SRR1952492	19,202,739	8,040,737 ; 1805,898	1h 51m 05s
	Tongue dorsum	SRR1944869	8,074,428	6,140,295 ; 499,284	1h 36m 58s
		SRR1952378	15,024,409	12,622,724 ; 891,920	3h 17m 30s
		SRR1952379	42,173,063	29,697,754 ; 2,084,990	7h 10m 23s
Vagina	Posterior fornix	SRR1951760	10,611,721	373,021 ; 24,484	42m 19s
		SRR1944797	8,242,829	120,519 ; 10,009	35m 14s
		SRR1944845	8,537,797	140,658 ; 10,779	34m 19s

Click here to access/download
Supplementary Material
SupplementaryMaterial.pdf

GIGA-D-17-00316
YAMP: a framework enabling reproducibility in metagenomics research
Tiphaine C Martin; Mario Falchi; Alessia Visconti
GigaScience

Dear Dr Edmunds,

We would like to thank you for your helpful comments, which we feel have substantially improved our manuscript. Please find in the following a point-by-point reply to your comments.
We hope that this new version of the manuscript is suitable for revision. Thank you for your time and consideration, we look forward to hearing from you soon regarding our submission.

1. For software, pipelines and workflows we always ask for comparisons to show it pushes the state-of-the-art, and that is the main issue that needs addressing here ans there are many more pipelines and tools out there.

Answer. We agree with the Editor that we overlooked the necessity of comparing the improvements brought by YAMP over other pipelines, limiting our discussion to a short paragraph within the Background Section. In the current version of the manuscript, we improved this discussion, highlighting the improvement brought by YAMP over the state-of-the-art pipelines. Briefly, MetAMOS and MOCAT2 do not support containerisation, while MG-RAST, the EBI metagenomic pipeline, and the workflows implemented within the Galaxy environment require users to upload their unpublished and/or confidential data on third-party servers, where they may wait several days to be processed. Galaxy could also be installed locally, but this has been shown to be potentially cumbersome (doi:10.3389/fcell.2014.00070) and could be undesirable when users necessitate only a few of the Galaxy modules and functionalities (such as those for metagenomics analysis alone). Additionally, YAMP includes QC steps not available within other pipelines, *e.g.*, decontamination, not included in the MetAMOS and the EBI metagenomic pipeline, and de-duplication, not included in the EBI metagenomic pipeline. The missing decontamination step allows reads not belonging to the studied ecosystem to be used in downstream analyses, potentially leading to erroneous read mapping on reference databases and functional profiling. Retaining duplicated reads, which are usually considered as technical artefacts derived from PCR amplification step (in library preparations which include PCR), may hamper the correct estimation of both the community composition and functional profile. Finally, despite MG-RAST performs de-duplication, this is performed after trimming. In our opinion, de-duplication should be performed beforehand, since changes in the read sequence due to trimming may hinder the identification of real duplicates.

2. Better justification is needed for your choice of tools and why you used nextflow.

Answer. We now include a new Section, called “Implementation”, which addresses these concerns. Tools from the BBmap suite, which we use for the QC steps, are widely used, computationally fast (thus scalable to large metagenomics projects) and able to handle both single- and paired-reads from the main sequencing platform. HUMAnN2 has been originally used to infer the functional and metabolic potential of microbial communities in the Human Microbiome Project, one of the largest of its kind, while MetaPhlAn2 has become the *de facto* standard when samples belong to well-characterised environments (doi:10.1038/nbt.3935). In the current version of the manuscript, we now stress that these are state-of-the-art tools, highlighting their strengths.
In the same section, we now detail the advantages Nextflow offers over other workflow management systems. First, Nextflow allows workflows to be both easy to develop and to use (doi:10.1093/bib/bbw020). This feature will be appreciated by both experienced

bioinformaticians, who will find easy to customise their analyses by introducing new steps, or modifying the existing ones, but also by novices, who can run their analyses using only a single command line instruction. Second, Nextflow offers user-transparent portability across a large number of platforms: from a personal computer, to an HPC cluster and AWS Amazon Cloud. Moreover, it also offers out-of-the-box support for the main cluster job schedulers (*e.g.*, SGE, LSF, SLURM, PBS/Torque), further improving its portability. Third, Nextflow offers implicit parallelisms, enabling a better use of computational resources and speeding up the analysis, ensuring YAMP scalability. Lastly, we believe that Nextflow is one of the best tool nowadays available to allow reproducibility, Indeed, it offers not only a seamlessly user-transparent support for Docker and Singularity (https://www.nextflow.io/docs/latest/docker.html#how-it-works), but it also for multiple code repositories (Bitbucket, GitHub, GitLab, https://www.nextflow.io/docs/latest/sharing.html). While the former allows tracking of the pieces of software used in the analyses and their versions, the latter allows software version control, resulting in a virtually perfect tracking of the version of the pipeline that has been run. Moreover, Nextflow provides a very detailed task execution report, which includes both the commands and the environment in with they were executed (*e.g.,* the working directory, the container, the used parameters). This feature helps to ensure YAMP retrospective provenance, and all these pieces of information are integrated within the very detailed YAMP log. We now include, in the YAMP GitHub wiki, a tutorial to help the users with the interpretation of the log file (https://github.com/alesssia/YAMP/wiki/How-to-read-the-logs), and where the users can also appreciate the details of the information stored.
Despite being officially published only a few months ago, these helpful and powerful Netxflow's features have been appreciated by several scientific developers, who have been using it to develop and sometime re-implement a number of pipelines both published (e.g., doi:10.1186/s12859-017-1770-1, doi: 10.1093/bioinformatics/btx709, doi: 10.1101/220616) and unpublished (*e.g.,* those listed at https://github.com/nextflow-io/awesome-nextflow).

3. And as the title goes it needs a bit more explanation why "yet another metagenomics pipeline" is needed, and why you are calling this a framework.

Answer: We defined YAMP as a framework to stress the possibility of generalising its backbone *(i.e.*, the Docker/Singularity integration with Nextflow) to support different research fields besides metagenomics. We, however, agree that it overstated, and we now call it "workflow", modifying the title to "YAMP: a containerised workflow enabling reproducibility in metagenomics research".
We also hope that the new pieces of information we included in the background section (and briefly described in the answer to this Editor's first point) will be helpful to understand why another metagenomics pipeline is actually needed.

3. Regarding the provenance claims, nextflow doesn't really have detailed tracking yet so a bit more explanation of how that works may be required.

Answer: We believe that the Nextflow's task execution reports (which includes, among the others, the exact command executed, the task working directory, environment and output, as well as the container image, as in https://www.nextflow.io/docs/latest/tracing.html and https://goo.gl/NpNUGS) paired with the YAMP configuration and log files, contain all the information necessary to ensure the so =-called *retrospective provenance* of each step executed in our pipeline, and, therefore, of the entire computational analysis. We now improved the discussion regarding provenance both when describing the advantages resulting from using Nextflow, and when describing YAMP output (that is now described in a separate section, called "YAMP Input/Output").

4. as does some discussion on maintenance and sustainability,

Answer: We understand the Editor's concerns regarding the maintenance and sustainability of our pipeline within the fast-developing metagenomics field. We developed YAMP for analysing the metagenomics samples that are and will be collected by multiple projects within our department, and therefore we will keep maintaining it, not only fixing issues that may arise but also expanding the analysis steps whether new analysis will be recognised as necessary, and keeping it updated with the state-of-the-art tools for the steps already available. Although the very first version of YAMP has been only very recently released, it has already attracted a number of users from both the human and animal metagenomics fields. Therefore, we have already addressed issues pointed out by YAMP users (*e.g.* https://github.com/alesssia/YAMP/issues/4). This easy maintenance is made feasible by the well-defined YAMP modularisation and the usage of standard data formats, which allow an easy update of the analysis flow and step -- helped by the ease of development ensured by Nextflow. These changes can be tracked through the GitHub repository using the Git versioning feature, ensuring the provenance requirements. Moreover, being an open source project, YAMP is open for community-driven improvement.
We now write: "In fact, the well-defined YAMP modularisation and the usage of standard data formats allow both an easy integration of new analysis steps and a customisation of the existing ones. This is of particular importance in a fast-developing field such as metagenomics, where the analysis guidelines and tools and are not stable yet. With YAMP we provide a framework to which new analysis modules can be easily added, and where tools that become outdated can be effortlessly replaced, therefore securing its sustainability.".

5. and why a container maintenance project like biocontainers was not used.

Answer: We opted for single container approach, as that offered by Docker, because we believe that, first, it is more agile when deploying the containerised workflow in an HPC cluster (the most likely deployment scenario for YAMP users), and second, it would be easier to handle by users with limited computational experience -- which will not need to deal with a multi-image scenario, as the one resulting using Biocontainer.
Regarding the first point, the Singularity technology (http://singularity.lbl.gov, doi:10.1371/journal.pone.0177459) is emerging as an effective alternative to Docker (of which it resolves the security concerns), and, in a multi-image scenario, users would need to manage and potentially convert to the Singularity format each container one by one, a process that is inconvenient, error-prone, and which increases the number of container images to be managed. This, along with obliging the inexperienced users to manage at least four different Biocontainer images would, in our opinion, undermine YAMP user-friendliness and would also allow for undocumented operations, consequently jeopardising the repeatability of the deployment procedure.

Personal Cover Click here to download Personal Cover coverLetter.pdf

Department of Twin Research & Genetic Epidemiology
King's College London
St Thomas' Hospital Campus
4thFloor South Wing Block D
Westminster Bridge Road
London
SE1 7EH

KING'S College LONDON

University of London

Dear Editor,

Please find enclosed our manuscript entitled *"YAMP: a containerised workflow enabling reproducibility in metagenomics research"* for consideration for publication as *Technical Note* in GigaScience.

In the manuscript, we present YAMP, a novel metagenomics pipeline, that processes raw shotgun metagenomics sequencing data up to the functional annotation. YAMP supports Docker and Singularity containerisation to ensure computational reproducibility and replicability, and to facilitate collaborative studies. YAMP can be executed on any UNIX-like system, and offers seamless support for multiple job schedulers as well as for Amazon AWS cloud.

YAMP has been developed to be extremely user-friendly and ready-to-use also by researchers with little bioinformatics or computational experience, and it is accompanied by a detailed documentation with several tutorials, and by a set of default parameters derived from our own analysis experience. However, the well-defined YAMP modularisation and the usage of standard data formats allow both an easy integration of new analysis steps and a customisation of the existing ones, a characteristic more experienced bioinformaticians will appreciate.

We believe that YAMP would help other researchers, especially if they are novices in the metagenomics or bioinformatics field.

Herein, we confirm that all authors have approved the manuscript for submission, and that the content of the manuscript has not been published, or submitted for publication elsewhere. We also declare having no potential competing interests.

Thank you for your time and consideration, we are looking forward to hearing back from you soon.

Best regards,
Dr Alessia Visconti

Telephone: +44 (0) 20 7188 5555
Fax: +44 (0) 20 7188 6717
Website: www.twinsuk.ac.uk
