# Supplementary material for: YAMP: a containerized workflow enabling reproducibility in metagenomics research
Source: Gigascience. 2018 Jun 18;7(7):giy072. doi: 10.1093/gigascience/giy072 (PMC6047416; doi:10.1093/gigascience/giy072)
Supplement: Supplement Files [file giy072_supplement_files.pdf]

Supplementary Material for "YAMP: a containerised workflow enabling reproducibility in metagenomics research"

Alessia Visconti[1], Tiphaine C. Martine,[1], and Mario Falchi,[1]
[1]Department of Twin Research and Genetic Epidemiology,
King's College London, London, UK

Supplementary Table S1: Simulated human oral community. The community includes 13 bacterial species. The genomic coverages, and, therefore, the relative proportion of the bacterial genomes, follow those suggested in Zhou Q, Su X, Ning K. *Assessment of quality control approaches for metagenomic data analysis.* Scientific reports (2014);4:6957 (`https://doi.org/10.1038/srep06957`), except for *Prevotella tannerae* which has been substituted with *Alloprevotella tannerae.* The reference microbial genomes were downloaded from the NCBI website (`http://www.ncbi.nlm.nih.gov`). We considered as *natural duplicates* the reads which were generated to match the requested coverage. † indicates that the GenBank assembly accession number has been reported.

**Species**	**RefSeq Accession number**	**%GC content**	**Genomic Coverage**	**Reads number**	**Reads percentage**	**Natural duplicates Reads (%)**
Rothia dentocariosa	GCF_002861025.1	53.75	6	149,659	22.80	8435 (5.64)
Porphyromonas endodontalis	GCF_000174815.1	47.52	5	103,225	15.73	2531 (2.45)
Streptococcus peroris	GCF_000187585.1	39.15	5	81,996	12.49	786 (0.96)
Alloprevotella tannerae	GCF_000159995.1	46.56	2	51,714	7.88	1501 (2.90)
Porphyromonas gingivalis	GCF_002892575.1	48.36	2	47,597	7.25	18 (0.04)
Veillonella atypica	GCF_001546845.1	38.88	2	41,977	6.40	521 (1.24)
Megasphaera micronuciformis	GCF_000165735.1	45.44	2	35,310	5.38	1101 (3.12)
Prevotella veroralis	GCF_000613325.1	41.80	1	28,344	4.32	294 (1.04)
Prevotella denticola	GCA_001062325.1†	50.05	1	25,990	3.96	20 (0.08)
Campylobacter rectus	GCF_000174175.1	44.85	1	25,131	3.83	133 (0.53)
Actinomyces odontolyticus	GCF_002847525.1	65.46	1	23,415	3.57	38 (0.16)
Veillonella parvula	GCF_002847925.1	38.57	1	21,505	3.28	248 (1.15)
Veillonella dispar	GCF_001553315.1	38.57	1	20,454	3.12	195 (0.95)

Supplementary Table S2: Simulated dataset. We report here the European Nucleotide Archive (ENA) project and sample accession numbers for the datasets used in the two simulation studies.

Simulated Study	Project Accession Number	Sample Accession Number	Setting
Human Contamination	PRJEB25791	ERS2327609	Baseline
		ERS2327610	1% contamination
		ERS2327611	5% contamination
		ERS2327612	25% contamination
		ERS2327613	50% contamination
		ERS2327614	80% contamination
Artificial Duplication	PRJEB26333	ERS2418541	Baseline
		ERS2418542	0.25% duplicates
		ERS2418543	1.25% duplicates
		ERS2418544	5.00% duplicates

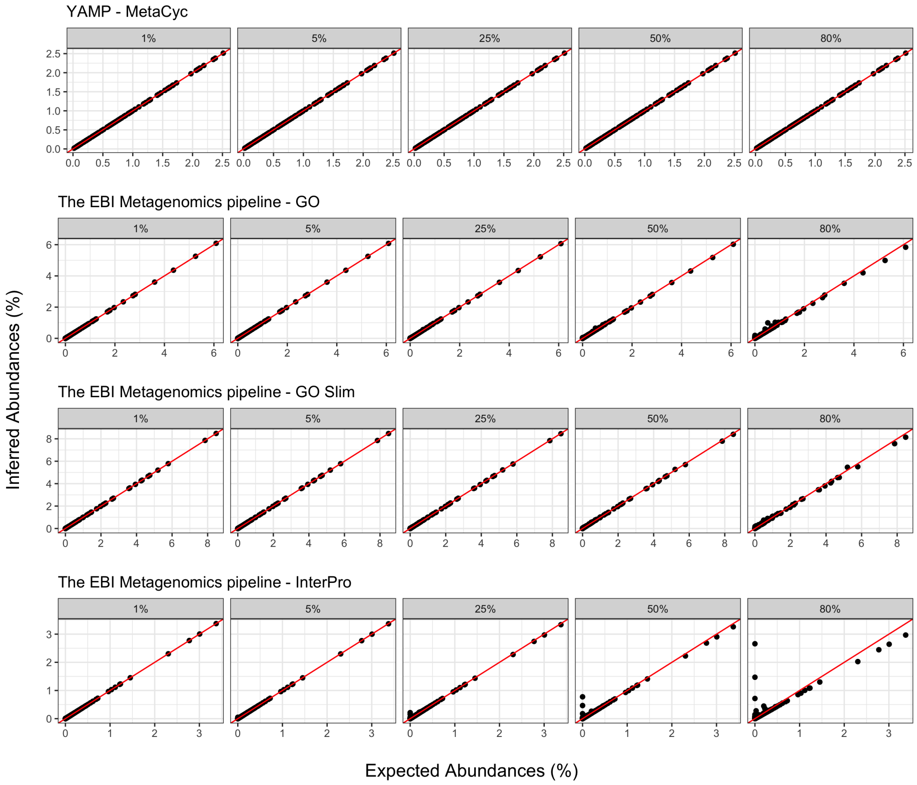

Supplementary Figure S1: Results of the first simulation study (human contamination). Each scatterplot represents the relationship between the expected functional annotations (*i.e.*, inferred without human contamination) and those inferred at different human contamination levels. For YAMP, we report results obtained with the MetaCyc database; for the EBI metagenomics pipeline (v4.1), we report results on the Gene Ontology (GO), GO Slim, and InterPro databases.

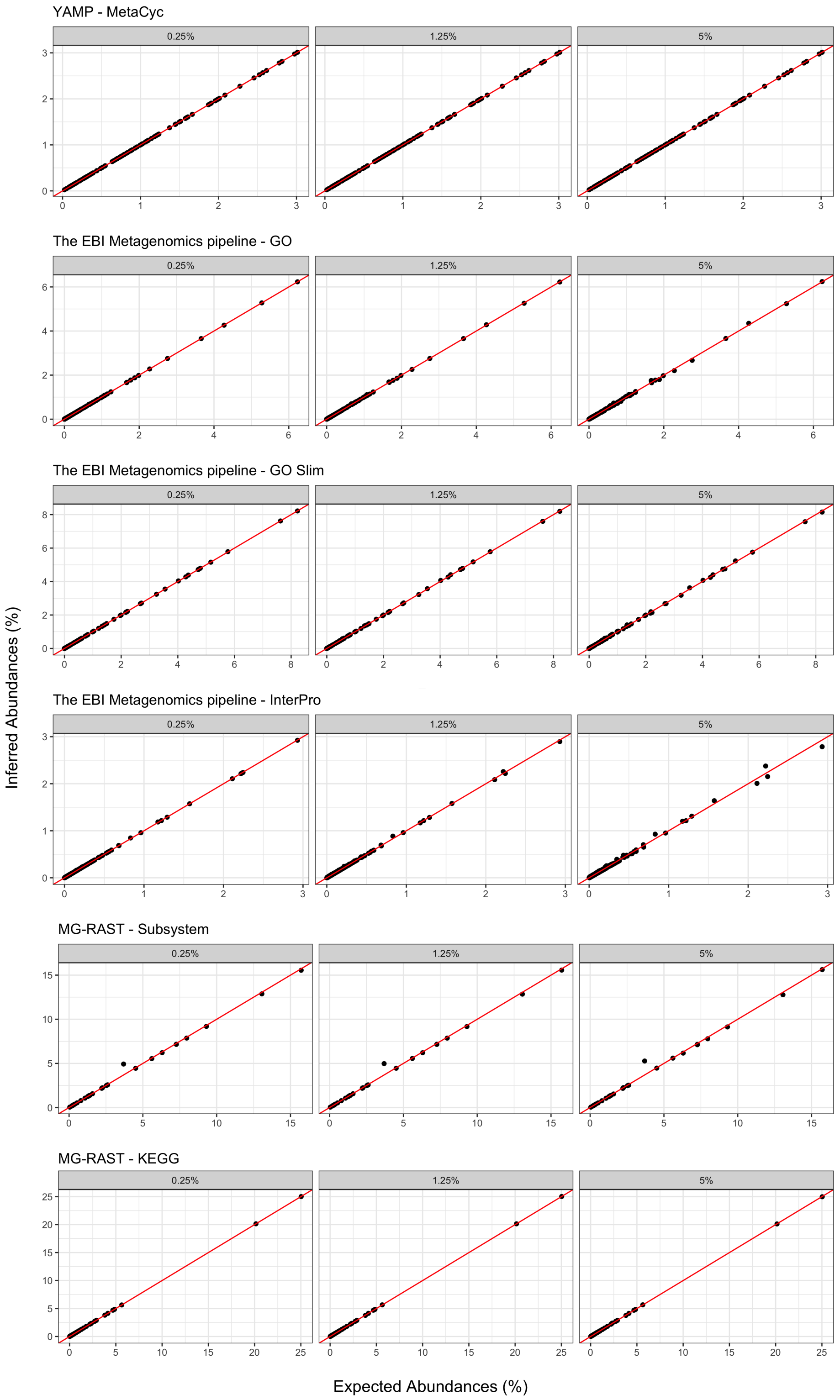

Supplementary Figure S2: Results of the second simulation study (artificial duplicates). Each scatterplot represents the relationship between the expected functional annotations (*i.e.*, inferred without duplicated reads) and those inferred at different levels of artificial duplication. For YAMP, we report results obtained with the MetaCyc database; for the EBI metagenomics pipeline (v4.1), we report results on the Gene Ontology (GO), GO Slim, and InterPro databases; for MG-RAST (v.4.0.3) we report results on the SEED Subsystem and the KEGG databases.

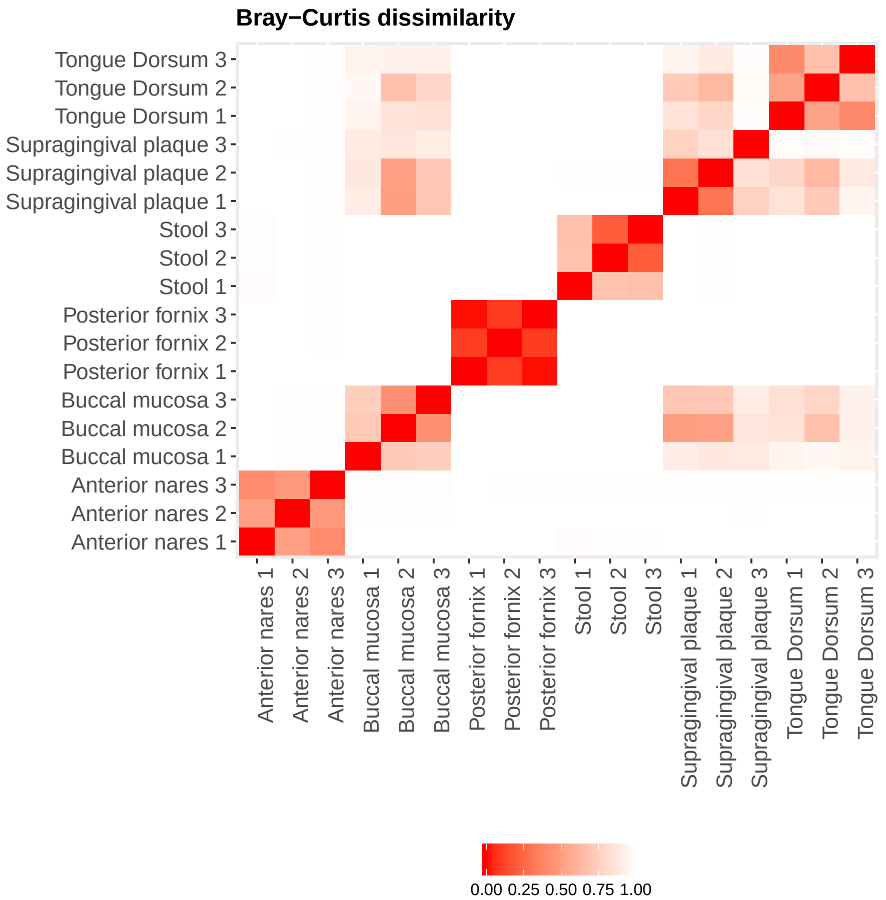

Supplementary Figure S3: Bray-Curtis dissimilarity for the 18 real-world samples. The Bray-Curtis dissimilarity values were evaluated using the species relative abundances as estimated by YAMP using MetaPhlAn2 and the *vegdist* function in the `vegan` R package (v2.4.3).

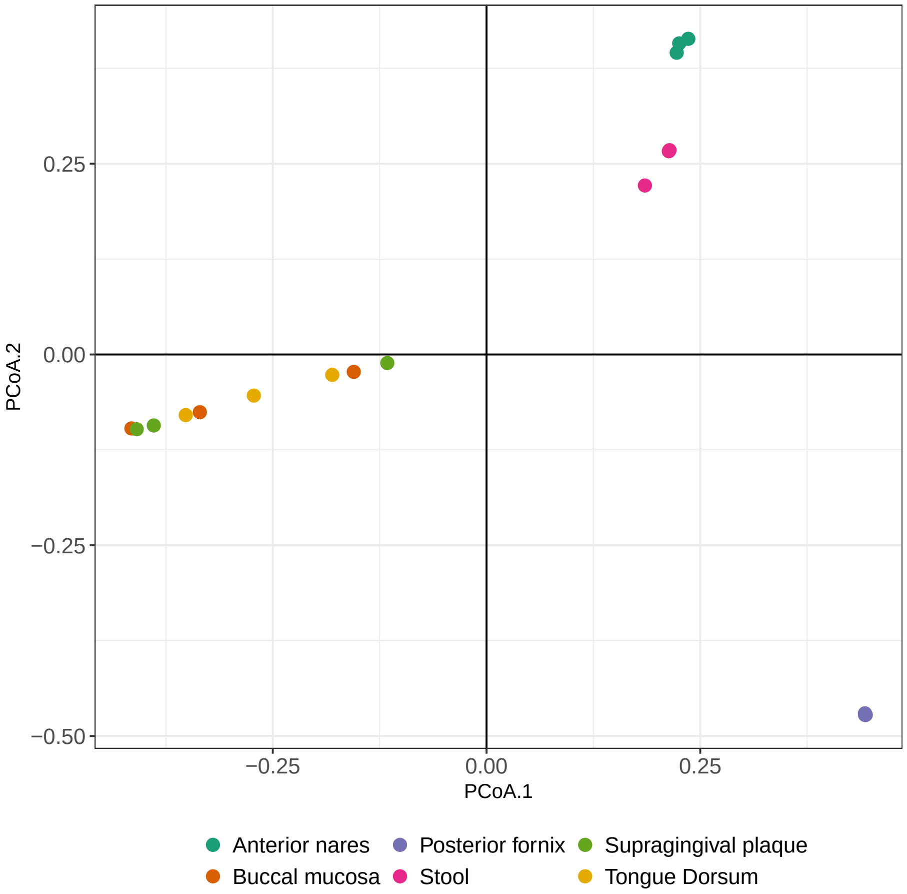

Supplementary Figure S4: Principal coordinate analysis (PCoA) for the 18 real-world samples. PCoA was evaluated on the Bray-Curtis dissimilarity values using the *pcoa* function in the `ape` R package (v4.1). PCoA shows that species composition is sufficient to discriminate among body sites, even though it has limited ability in distinguishing among different loci in the oral cavity.

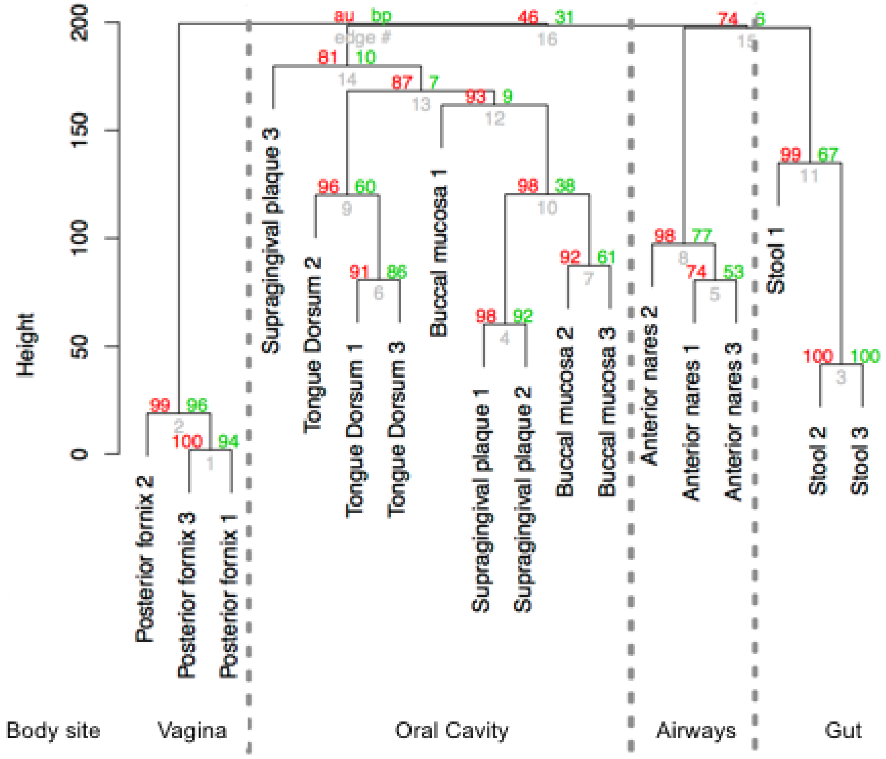

Supplementary Figure S5: Hierarchical clustering for the 18 real-world samples. Hierarchical clustering was computed using the Manhattan distance among species relative abundances using the *pvclust* function in the `pvclust` R package (v2.0). Values at branches are, in red, the approximately unbiased (AU) P values, in green, the bootstrap probability (BP) values (percentages), and, in grey, the edge number. The clustering shows that species composition is sufficient to discriminate among body sites.
