# Supplementary material for: YAMP: a containerized workflow enabling reproducibility in metagenomics research
Source: Gigascience. 2018 Jun 18;7(7):giy072. doi: 10.1093/gigascience/giy072 (PMC6047416; doi:10.1093/gigascience/giy072)
Supplement: Response_to_Reviewer_Comments_Original_Submission.pdf [file giy072_response_to_reviewer_comments_original_submission.pdf]

Author's Response To Reviewer Comments

Close

GIGA-D-18-00004:
YAMP: a containerised workflow enabling reproducibility in metagenomics research,
Alessia Visconti; Tiphaine C Martin; Mario Falchi

Dear Dr Edmunds,
We would like to thank you and the Reviewers for your helpful comments. We really appreciated the time spent to perform such a complete and detailed revision.
Please find in the following a point-by-point reply to the comments. We have also tracked all changes in the revised manuscript using a red font-face.
Thank you for your time and consideration, we look forward to hearing from you soon regarding our submission.

Kind regards,
Dr Alessia Visconti

Editor's comments:

1. Your manuscript "YAMP: a containerised work flow enabling reproducibility in metagenomics research" (GIGA-D-18-00004) has been assessed by our reviewers. Although it is support of interest, we are unable to consider it for publication in its current form. The reviewers have raised a number of points which we believe would improve the manuscript and may allow a revised version to be published in GigaScience. In particular we require testing and comparisons against the state-of-the art in our software/technical note papers so make sure this is sufficiently addressed, as well as issues about long-term support and stability (the biocontainers point has been raised again, and CWL may help).

Answer: We have performed a comparative study in two different simulated scenarios to assess YAMP's performances against both the EBI metagenomics pipeline and MG-RAST. Please refer to the answer to Reviewer #2 for more details. Also, we have now introduced and described a multi-container scenario, which could be of use for more expert users which may want/need to customize YAMP or to explore different tools. Please refer to the answer to Reviewer #1 for more details.

2. Another published pipeline you might want to cite is:
http://genome.cshlp.org/content/19/11/2144.long

Answer: We are citing "Pond, Sergei Kosakovsky, et al. Windshield splatter analysis with the Galaxy metagenomic pipeline." Genome research 19.11 (2009): 2144-2153" as an example of metagenomics platform available within the Galaxy framework in the "Introduction" section (ref 22).

3. In addition, please register any new software application and workflows in the SciCrunch.org database to receive a RRID (Research Resource Identification Initiative ID) number, and include this in your manuscript. This will facilitate tracking, reproducibility and re-use of your tool.

Answer: We have registered YAMP in the SciCrunch database, and now report the RRID in the manuscript:
“Here we present “Yet Another Metagenomic Pipeline" (YAMP), a ready-to-use containerised workflow that, using state-of-the-art tools, processes raw shotgun metagenomic sequencing data up to the taxonomic and functional annotation (Scicrunch.org RRID: SCR_016236)”

4. And please update the bioRxiv preprint as this cannot be carried out after publication. As a FYI we are now displaying this among under consideration preprints here,but let us know if you have any problems with that:
http://preprint.space/byjournal/gigascience?published=0

Answer: We have updated the bioRxiv preprint to match the revised manuscript now submitted. It has been assigned the ID “BIORXIV/2017/223016”. We are happy with the fact that our preprint is listed among those under consideration by GigaScience.

Reviewers' comments:

Reviewer #1

The authors describe a well designed metagenomic analysis pipeline, integrating a set of previously available bioinformatics tools through the NextFlow workflow execution framework. The pipeline is open source and the code along with documentation is available at https://github.com/alesssia/YAMP
The one concern I have as reviewer and bioinformatics researcher, is with the implementation and the claims of the authors for a "user-friendly". Definitely, integrating the tools to create an complete analysis pipeline in NextFlow provides great convenience, but it would still not be accessible to end-users (since Nextflow is command-line only). For example, to be user-friendly as claimed, the authors could have implemented the pipeline in Galaxy, which would have provided full accessibility to end-users given the web-based, intuitive interface of Galaxy.
Another example for this, the authors compare with CLOVR metagenomics, but the CLOVR is a fully self-contained solution with in a Virtualbox or Amazon cloud virtual machine, and the users of CLOVR do not have to do any installation.
An example of all-integrated, user-friendly and Docker-based pipeline for bioinformatics pipelines is the following recent publication from our group on this journal: Kim B, Ali T, Lijeron C, Afgan E, Krampis K. Bio-Docklets: virtualization containers for single-step execution of NGS pipelines. GigaScience. 2017 Aug 1.
Based on these facts, and while I support the author's choice of Nextflow since has advantages over Galaxy for scalability, the following need to be improved in the paper before publication, mainly from the perspective of less-technically adept users, but also for bioinformatics developers who will try to utilize the pipeline:

Answer: As correctly pointed out by the Reviewer, we preferred Nextflow also because of its scalability advantages. Moreover, while MG-RAST, the EBI metagenomics pipeline, and the workflows implemented within the Galaxy environment require users to upload their data on third-party servers, where they may wait several days to be processed, YAMP can run on dedicated platforms (either local computational facilities, or cloud services) without

delays. During the comparative study we carried out (please see the answer to Reviewer #2 for more details), and which involved testing the performances of both the EBI metagenomics pipeline and MG-RAST, we noticed that the former required about five days from the time of data submission to the availability of the analysis results, while the latter required up to 11 days. In both cases, most of the time was spend in the waiting queue.
We are aware that Galaxy could also be installed locally, but this has been shown to be potentially cumbersome (doi:10.3389/fcell.2014.00070) and could be undesirable when users necessitate only a few of the Galaxy modules and functionalities (such as those for metagenomics analysis alone).
We also argue that the installation and configuration of Virtualbox, or the deployment of AWS necessary to use CLOVR may not be straightforward, and users with no knowledge of these technologies may find it difficult to have the system up and running. We are aware that the usage of the command line may be scaring for many users, however it allows portability of the workflow also on platforms which do not support Graphical User Interfaces, as most of the HPC facility available within research centers. Bio-Dockets itself require the usage of the command line to be started (https://github.com/BCIL/BioDocklets).
Finally, the set of command line instructions that are necessary for setting up and running YAMP is very limited (and available within a new tutorial that can be found in the GitHub YAMP wiki: https://github.com/alesssia/YAMP/wiki/TL%3BDR, please refer also to the answer to your point #2).

1. According to the documentation (https://github.com/alesssia/YAMP) the following dependencies are required to be installed and configured by the users: fastQC, BBmap, Samtools, MetaPhlAn2, QIIME, HUMAnN2. Furthermore while the NextFlow is a .jar file, it still might need a java distribution installed on the user's system which is not mentioned as dependency, and additionally Docker should be mentioned as dependency as well. My request to the authors is that since NextFlow has build-in the capacity to run a tool that is preinstalled in a container, the tools mentioned as required dependencies should be provided pre-installed within containers, and called as such from within the pipeline. In this way, the bioinformatics developers who will try to use the pipeline would not have to go through installing all these dependencies (which would take significant amount of time even for a developer, and might be impossible for a non-command line expert user).

Answer: We are sorry that the “Dependencies” section on the YAMP GitHub page generated this confusion. Indeed, YAMP is already accompanied by a container with includes all the software required in the pipeline execution and that is fully compatible with both Docker and Singularity, allowing users to run their analyses without having to install any software but Nextflow, Docker, and/or Singularity. We had also pointed out that these pieces of software should be installed separately, along with JAVA and BASH, linking to the relevant web pages, and also indicating known Java version incompatibility. We have now made this clearer, since we agree with the Reviewer that this confusion may alarm users. The Reviewer will find the updated version on both the YAMP main GitHub page (https://github.com/alesssia/YAMP) and in the wiki (https://github.com/alesssia/YAMP/wiki/Getting-started). If the Reviewer would like to see the tracked difference he can do so, for instance at the following address: https://github.com/alesssia/YAMP/commit/6beaff3865ff353245ced337d85ce33a06438d1c#diff-04c6e90faac2675aa89e2176d2eec7d8. To further clarify this, we added Java as a dependence also in the “Availability of source code and requirements” Section.
The authors might not even have to create containers with these tools pre-installed, since most possibly the repositories such as BioContainers (https://biocontainers.pro/) and

BioShadock (https://docker-ui.genouest.org/app/#/) might have containers with these dependencies tools among the tens of containers listed there, The authors simply need to specify the repository / container address in the NextFlow code, and during execution the NextFlow engine will pull and deploy automatically these containers in the user's system during runtime.

Answer: We opted for single container approach, as that offered by Docker/Singularity and seamlessly supported by Nextflow and that we made available in DockerHub (https://hub.docker.com/r/alesssia/yampdocker/), because we believe that, first, it is more agile when deploying the containerised workflow in an HPC cluster (the most likely deployment scenario for YAMP users), and second, it would be easier to handle by users with limited computational experience -- which will not need to deal with a multi-image scenario, as the one resulting using, for instance, Biocontainer. However, we agree that a multi-container scenario may be useful for more expert users who may want/need to customize YAMP or to explore different tools. We now describe this scenario in a dedicated tutorial (https://github.com/alesssia/YAMP/wiki/multi-image-scenario) and add write in the manuscript
("Implementation" Section):
"YAMP is accompanied by a Docker and a Singularity [30] container. While Docker defines a platform-independent virtualised light-weight operating system that includes all the pieces of software required by YAMP and traces their versioning, Singularity allows these features to be transferred to High Performance Computing (HPC) systems, with which Docker is inherently incompatible. Along with this single-container approach, YAMP also supports a multi-container scenario. Indeed, while the former is of easier management for users with limited computational experience and allows a more agile deployment, the latter accommodates for YAMP customisation without losing the advantages of a containerised solution in terms of reproducibility and ease of setup."

2. Provide user documentation as a supplementary file, explaining the basic commands to run the pipeline, the directory structure needed to be created by the user in order for the pipeline to execute without error, in addition to explaining where to place within this pipeline run directory structure the required supporting files (FASTA with adapter, contaminating genomes, BowTie2, ChocoPhlan databases) mentioned on the documentation (https://github.com/alesssia/YAMP)

Answer: We favorited providing an on-line user documentation via the GitHub platform as a wiki webpage (https://github.com/alesssia/YAMP/wiki). This allowed and will allow us to accommodate for changes and upgrades, further ensuring that users will always be provided with the most updated documentation. In the wiki pages, users could already find a tutorial on how to run YAMP (https://github.com/alesssia/YAMP/wiki/How-to-run-YAMP), which included also an example of the folder layout (https://github.com/alesssia/YAMP/wiki/Folders-layout-example). Notably, users do not have to follow a strict folder layout and can specify the path of the requested resources in the nextflow.config file as explained in the https://github.com/alesssia/YAMP/wiki/How-to-run-YAMP tutorial. Remarkably, using different folder layout will not jeopardise reproducibility since each resource path is printed in the YAMP log file, thus ensuring retrospective provenance. We now stress this flexibility version on both the YAMP main GitHub page (https://github.com/alesssia/YAMP) and in the wiki (https://github.com/alesssia/YAMP/wiki/Folders-layout-example, https://github.com/alesssia/YAMP/wiki/How-to-run-YAMP,

https://github.com/alesssia/YAMP/wiki/Getting-started). Following the Reviewer's suggestion, we have also created an additional tutorial, which accompanies the more detailed https://github.com/alesssia/YAMP/wiki/How-to-run-YAMP already available, and provides a simplified (and shorter) guidance for the user. This tutorial, called "TL;TR" can be find here: https://github.com/alesssia/YAMP/wiki/TL;TR.
We now highlight these online resources reporting the wiki link in the manuscript, where we write:
"The source code is available in the YAMP GitHub repository (https://github.com/alesssia/YAMP), which includes a wiki with a full documentation and several tutorials (https://github.com/alesssia/YAMP/wiki).

3. Provide space and time metrics for a run of the pipeline within a specific computational setup, with the datasets mentioned as example in the manuscript.

Answer: We have provided the running time figures in Table 3, which were generated using on a machine sporting a 2.60GHz Intel® Xeon® processor with 32 GB of RAM. However, we did not report that these figures were generated using the default configuration setup, described in the nextflow.config file. We now edited the manuscript as:
"On average, the selected samples included 12.6M paired-end reads (25.2M reads in total), which yielded to 13.3M QC'ed reads (including both paired-end and singleton reads), and were processed in an average time of two hours using four threads on a machine sporting a 2.60GHz Intel® Xeon® processor with 32 GB of RAM and using the default YAMP parameters (Table 3)."
We thank the Reviewer for pointing out the we did not mention the disk space necessary to run YAMP. This is indeed a crucial information for successfully carry out the data analysis. In fact, the disk space requested by the files generated through this pipeline is, in average, about seven times the size of the original samples, and the problem will exacerbate when multiple samples are processed simultaneously, as in multicore machines or HPC facilities. By using the keepQCtmpfile and the keepCCtmpfile parameters, the users can decide whether or not to save the temporary files generated during the YAMP execution and therefore decreasing the disk space occupancy. When temporary files are not saved, indeed, the disk space required is about 20-70% smaller than the size of the original samples, a value that is strongly dependent of the samples' quality. In fact, the only large file generated by YAMP is the FASTQ file of QC'ed reads, and the higher the starting data quality the bigger the QC'ed reads file size, since little reads will be discarded. The other files (namely the QC reports, the taxonomy composition, the microbe, gene and pathway relative abundances, the pathway coverage, the α-diversity measures, and the logs) usually only occupies tens of MB.
We now add these statistics in last column of Table 3 and write in the manuscript ("Input/output" Section):
"It should be noted that the disk space requested by the files generated by YAMP is, in average, about seven times the size of the raw data files, and particular attention should be paid when multiple samples are processed simultaneously, as in multicore machines or HPC facilities. However, discarding temporary files, as we suggest, will require a final disk space of 20-70% the size of the raw data files, with higher quality files requiring more space due to the small number of reads discarded during the QC process."

Ideally, since Amazon Web Services (AWS) is mentioned as a platform where the pipeline can run, the authors should demonstrate that. In fact, it would be a great benefit for the intended audience if the authors set up an AWS virtual machine image with all requirements

pre-installed and make it publicly available along with the code (users would run it on their own expense under their Amazon account).

Answer: AWS support is guaranteed by Nextflow, which smoothly integrates with AWS Batch. AWS Batch allows the execution of containerised workloads over the Amazon EC2 Container Service (ECS), supporting auto-scaling ability and the use of spot instances making the use of the AWS system more cost effective.
We agree with the Reviewer that offering a pre-build AWS image would benefit users, however, we decided against it for two reasons. First, YAMP has not been designed with a specific metagenomics environment in mind, and, while we provided the resources necessary for analyzing human metagenomics data (such as the human masked FASTA file for decontaminating our samples), we cannot generate all the supporting dataset/databases that could be necessary for each specific analysis. For instance, we already have users which used YAMP to analyse mouse and soil samples (see here: https://github.com/alesssia/YAMP/issues/4 and here: https://github.com/alesssia/YAMP/issues/6), and these analyses will require custom genomes for decontamination, and very specific and/or manually curated databases for functional annotation. Second, we have no budget allocated for AWS resources due to the fact that our laboratory preferred investing on our dedicated physical computer server, which proved to be a cheaper way to run our experiments. We also believe this to be the most common scenario for our users.
However, we are convinced that the deployment of YAMP on the cloud allows flexibility and scalability not possible with common on-premises clusters, and that some research groups may prefer to pay for leasing computing time and space on the cloud rather than invest on a big in-house server and its operational and maintenance costs. For this reason, we created a new tutorial on the YAMP wiki (https://github.com/alesssia/YAMP/wiki/How-to-use-AWS-Batch) which explain how to integrate YAMP with AWS Batch, and how to upload/link the specific resources required.

Reviewer #2:

The article underlines the significance of research reproducibility and how the state-of-the-art containerization methodologies could be used, in order to overcome the respective obstacles. Keeping users away from installing a plethora of tools and checking libraries compatibility, containerization provides black-box solutions, in order to execute complex algorithmic tasks, but also an isolated environment that can be customized by the user. The proposed workflow, which is also distributed in containerized version, could have beneficial impact in the scientific community, providing a unified operator about metagenomics analysis and indicating a modern way to enhance repeatability in science and data analysis. Thus, the majority of comments concern to the core of metagenomics pipeline and not to the concept of virtualization.

1. It would be useful to mention if there are other published containerized bioinformatics workflows (not only in metagenomics), or the proposed work is actually pioneering.

Answer: We are aware only of two other containerised workflows that has been implemented with the idea of combining a workflow development framework with a container technology, that is the Integrated Meta-omic Pipeline (IMP, https://doi.org/10.1186/s13059-016-1116-8) and Bio-Docklets

(https://doi.org/10.1093/gigascience/gix048). However, bioinformaticians starts to appreciate the advantages of such solution and many are the re-implementation of existing pipelines to support containerization (for instance, the GATK RNA-Seq Variant Calling and the Kallisto & Sleuth RNA-Seq Tools, https://github.com/CRG-CNAG/CalliNGS-NF, https://github.com/cbcrg/kallisto-nf).
We know write, in the "Potential implications" Section:
"YAMP, along with other containarised workflows, such as the Integrated Meta-omic Pipeline (IMP) [58] and Bio-Docklets [59], represents a proof-of-concept showing a simple way to enable reproducible and collaborative research."

2. In "Implementation" section, a justification for the choice of QIIME pipeline is important, as you sufficiently performed for all the other methodologies/tools. Also a short description about QIIME pipeline's functions that are used in YAMP is needed.

Answer: We agree with the Reviewer that the choice of QIIME is not well justified, and now write in the "Implementation" Section:
"The identified taxonomic profile is additionally used by YAMP to evaluate multiple α-diversity measures through the alpha_diversity.py function available within the widely-used QIIME pipeline [43]. QIIME is an extremely modular and efficient pipeline designed for the analysis of amplicon (e.g., 16S or 18S rRNA genes) sequencing data, which also implements a number of functions investigating ecological features relevant in metagenomics research."

3. "Input/Output" section may requires a clarification about how you define the set of default parameters (what reads length you used, how strict was the analysis, thresholds-errors tradeoff).

Answer: We agree with the Reviewer that the default parameters were not discussed, and that the rationale behind them is of utmost importance if the users need to modify them according to their own needs and analysis. We now write in the "YAMP Input/Output" Section:
"More in details, we suggest retaining bases with a Phred score of at least 10 (Q10), representing a base call accuracy of 90%, i.e., the probability of calling a base out of ten incorrectly. This allows the retrieval also of low coverage regions, therefore improving the total genome recovery and contiguity, an aspect of utmost importance when the QC reads are used for assembly. We also recommend to conservatively discard all reads shorter than 60 bp after trimming, corresponding to a complexity of 460 or less. This length is considered the lowest for avoiding spurious signals when carrying on functional characterisation via HUMAnN2 [44, 45 46]. Next, we propose using a minimum alignment identity of 95% (maximum indel length: 3 bp) to identify contaminants reads, which has been shown to possibly zero the number of false positive when used on opportunely created custom database of contaminant reads [47]. Finally, we suggest using the UniRef90 protein database, as its clusters are more likely to be iso-functional and non-redundant. However, the UniRef50 protein database should be preferred when dealing with poorly characterised microbiomes."

4. You describe the disadvantages of other metagenomics analysis workflows, however there is not any comparison in "Results" section (time & resources consuming or quality control effectiveness). It would be notable to verify some of your introductory comments with a comparative analysis.

Answer: We recognize that this is a very important point, and now carry out a comparative study versus other state-of-the-art metagenomics workflows. Among those cited in the Introduction of the manuscript, we decided to select only workflows which offered the same advantages offered by YAMP, that is containerisation and a complete QC. Indeed, MetAMOS, MOCAT2, and RAMMCAP do not support containarisation, while CloVR-Metagenomics does not perform any QC step, and allows processing reads only generated with the Roche 454 pyrosequencing platform. This leave us with the EBI metagenomics pipeline and MG-RAST. We focused in assessing mostly the effectiveness of the quality control step and how it affects the functional characterization of the microbial community, which we name as the main disadvantages when compared with YAMP. We avoided a comparison on time and resources requirements since both the EBI metagenomics pipeline and MG-RAST allow analyses to be carried out on specifically designed third-party servers (therefore both requiring virtually no resources and being highly dependent on the specific workload at the time of job submission). We, however, observed that, while the EBI metagenomics pipeline required a quite stable analysis time of five days between sample upload and availability of the results, the analysis time for MG-RAST varied between few hours and 11 days, with most of this time spend in the waiting queue, as anticipated.
More in details, we carried out two simulation studies which used eight simulated datasets, including, in the first study, different percentages of human contamination (from 1 to 80%, as observed in the literature) and, in the second study, of duplicated reads (from 0.25 to 5%, again, as already observed). In the first simulation study, we compared the functional profiles inferred by YAMP with those generated using the EBI metagenomics pipeline (version 4.1), which does not include a decontamination step. In the second study, we compared the functional profiles generated by YAMP with both those generated using the EBI metagenomics pipeline (version 4.1), which does not include a de-duplication step, and those generated using the MG-RAST pipeline (version 4.0.3), which performs trimming before de-duplication, therefore, in our opinion, introducing biases. In both simulation studies, the results obtained by YAMP outperform those obtained by its competitors, even though the EBI metagenomics pipeline offers good results, especially at low levels of human contamination and artificial duplication.
The simulated datasets are available from the European Nucleotide Archive website (Study accession numbers: PRJEB25791 and PRJEB26333).
We now extended the Introduction, Results, and Methods sections to explain the design of the simulation studies, and to describe the obtained results. The Reviewer can find these novel parts of the manuscript highlighted with a red font-face in the revised manuscript.

Close
