# Supplementary material for: YAMP: a containerized workflow enabling reproducibility in metagenomics research
Source: Gigascience. 2018 Jun 18;7(7):giy072. doi: 10.1093/gigascience/giy072 (PMC6047416; doi:10.1093/gigascience/giy072)
Supplement: Response_to_Reviewer_Comments_Revision_1.pdf [file giy072_response_to_reviewer_comments_revision_1.pdf]

Author's Response To Reviewer Comments

Close

GIGA-D-18-00004R1:
YAMP: a containerised workflow enabling reproducibility in metagenomics research,
Alessia Visconti; Tiphaine C Martin; and Mario Falchi

Dear Dr Edmunds,
We would like to thank again you and the Reviewers for your comments.
Please find in the following a point-by-point reply to your comments.

Thank you for your time and consideration,
Kind regards,
Dr Alessia Visconti

Editor's comments:

Your manuscript "YAMP: a containerised workflow enabling reproducibility in metagenomics research" (GIGA-D-18-00004R1) has been assessed by our reviewers. Based on these reports, and my own assessment as Editor, I am pleased to inform you that it is potentially acceptable for publication in GigaScience, once you have carried out some final formatting changes:
You need to add an "Availability of supporting data" section just before the "Availability of Source Code and Requirements" and cite the data here (probably as reference 63). This can say something like this:
Availability of supporting data
Snapshots of the sourcecode are available from the GigaScience GigaDB repository[63].
You can then cite the data as follows:
Visconti, A; Martin, T, C; Falchi, M (2018): Supporting data for "YAMP: a containerised workflow enabling reproducibility in metagenomics research" GigaScience Database.

Answer: We have now included a new section called "Availability of supporting data" where we cite the datasets and the snapshot of the source code as suggested. More specifically, we write:
"Snapshots of the source code are available from the GigaScience GigaDB repository [67]. The Docker/Singularity image can be downloaded and installed from DockerHub [68]. The databases queried by YAMP during the simulation and real-world case studies are available from Zenodo [63]."

And you might also want to cite the Zenodo data here too, and go back through the paper and cite relevant URLs in the references as the production team will ask you to do this.

Answer: We have cited the Zenodo data (as well as the Docker image) in the Section "Availability of supporting data". We have also cited all the URLs in the references rather than in the main text.

You can also add the RRID to the "Availability of Source Code and Requirements" section

as well.

Answer: We have included the RRID and the link to YAMP Documentation and tutorials in the “Availability of Source Code and Requirements".

Close
