# Supplementary material for: YAMP: a containerized workflow enabling reproducibility in metagenomics research
Source: Gigascience. 2018 Jun 18;7(7):giy072. doi: 10.1093/gigascience/giy072 (PMC6047416; doi:10.1093/gigascience/giy072)
Supplement: Reviewer_1_Report_(Original_Submission) -- Konstantinos Krampis, PHD — 01/28/2018 Reviewed [file giy072_reviewer_1_report_(original_submission).pdf]

Reviewer Report

Title: YAMP: a containerised workflow enabling reproducibility in metagenomics research

Version: Original Submission **Date:** 1/28/2018

Reviewer name: Konstantinos Krampis

Reviewer Comments to Author:

The authors describe a well designed metagenomic analysis pipeline, integrating a set of previously available bioinformatics tools through the NextFlow workflow execution framework. The pipeline is open source and the code along with documentation is available at https://github.com/alesssia/YAMPThe one concern I have as reviewer and bioinformatics researcher, is with the implementation and the claims of the authors for a "user-friendly". Definitely, integrating the tools to create an complete analysis pipeline in NextFlow provides great convenience, but it would still not be accessible to end-users (since Nextflow is command-line only). For example, to be user-friendly as claimed, the authors could have implemented the pipeline in Galaxy, which would have provided full accessibility to end-users given the web-based, intuitive interface of Galaxy. Another example for this, the authors compare with CLOVR metagenomics, but the CLOVR is a fully self-contained solution with in a Virtualbox or Amazon cloud virtual machine, and the users of CLOVR do not have to do any installation.An example of all-integrated, user-friendly and Docker-based pipeline for bioinformatics pipelines is the following recent publication from our group on this journal: Kim B, Ali T, Lijeron C, Afgan E, Krampis K. Bio-Docklets: virtualization containers for single-step execution of NGS pipelines. GigaScience. 2017 Aug 1.Based on these facts, and while I support the author's choice of Nextflow since has advantages over Galaxy for scalability, the following need to be improved in the paper before publication, mainly from the perspective of less-technically adept users, but also for bioinformatics developers who will try to utilize the pipeline: 1. According to the documentation (https://github.com/alesssia/YAMP), the following dependencies are required to be installed and configured by the users: fastQC, BBmap, Samtools, MetaPhlAn2, QIIME, HUMAnN2. Furthermore while the NextFlow is a .jar file, it still might need a java distribution installed on the user's system which is not mentioned as dependency, and additionally Docker should be mentioned as dependency as well. My request to the authors is that since NextFlow has build-in the capacity to run a tool that is preinstalled in a container, the tools mentioned as required dependencies should be provided pre-installed within containers, and called as such from within the pipeline. In this way, the bioinformatics developers who will try to use the pipeline would not have to go through installing all these dependencies (which would take significant amount of time even for a developer, and might be impossible for a non-command line expert user). The authors might not even have to create containers with these tools pre-installed, since most possibly the repositories such as BioContainers (https://biocontainers.pro/) and BioShadock (https://docker-ui.genouest.org/app/#/) might have containers with these dependencies tools among the tens of containers listed there, The authors simply need to specify the repository / container address in the NextFlow code, and during execution the NextFlow engine will pull and deploy automatically these containers in the user's system during runtime. 2. Provide user documentation as a supplementary file, explaining the basic commands to run the pipeline, the directory structure needed to be created by the user in order for the pipeline to execute without error, in addition to explaining where to place within this pipeline run directory structure the required supporting files (FASTA with adapter, contaminating genomes, BowTie2, ChocoPhlan databases) mentioned on the documentation (https://github.com/alesssia/YAMP).3. Provide space and time metrics for a run of the pipeline within a specific computational setup, with the datasets mentioned as example in the manuscript. Ideally, since Amazon Web Services (AWS) is mentioned as a platform where the pipeline can run, the authors should demonstrate that. In fact, it would be a great benefit for the intended audience if the authors set up an AWS virtual machine image with all requirements pre-installed and make it publicly available along with the code (users would run it on their own expense under their Amazon account).

Level of Interest

Please indicate how interesting you found the manuscript: An article whose findings are important to those with closely related research interests

Quality of Written English

Please indicate the quality of language in the manuscript: Acceptable

Declaration of Competing Interests

Please complete a declaration of competing interests, considering the following questions:

- Have you in the past five years received reimbursements, fees, funding, or salary from an organisation that may in any way gain or lose financially from the publication of this manuscript, either now or in the future?
- Do you hold any stocks or shares in an organisation that may in any way gain or lose financially from the publication of this manuscript, either now or in the future?
- Do you hold or are you currently applying for any patents relating to the content of the manuscript?
- Have you received reimbursements, fees, funding, or salary from an organization that holds or has applied for patents relating to the content of the manuscript?
- Do you have any other financial competing interests?
- Do you have any non-financial competing interests in relation to this paper?

If you can answer no to all of the above, write 'I declare that I have no competing interests' below. If your reply is yes to any, please give details below.

No competing interests.

I agree to the open peer review policy of the journal. I understand that my name will be included on my report to the authors and, if the manuscript is accepted for publication, my named report including any attachments I upload will be posted on the website along with the authors' responses. I agree for my report to be made available under an Open Access Creative Commons CC-BY license (http://creativecommons.org/licenses/by/4.0/). I understand that any comments which I do not wish to be included in my named report can be included as confidential comments to the editors, which will not be published.

I agree to the open peer review policy of the journal
