# Supplementary material for: YAMP: a containerized workflow enabling reproducibility in metagenomics research
Source: Gigascience. 2018 Jun 18;7(7):giy072. doi: 10.1093/gigascience/giy072 (PMC6047416; doi:10.1093/gigascience/giy072)
Supplement: Reviewer_2_Report_(Original_Submission) -- Theodore Koutsandreas — 02/01/2018 Reviewed [file giy072_reviewer_2_report_(original_submission).pdf]

Reviewer Report

Title: YAMP: a containerised workflow enabling reproducibility in metagenomics research

Version: Original Submission **Date:** 2/1/2018

Reviewer name: Theodore Koutsandreas

Reviewer Comments to Author:

The article underlines the significance of research reproducibility and how the state-of-the-art containerization methodologies could be used, in order to overcome the respective obstacles. Keeping users away from installing a plethora of tools and checking libraries compatibility, containerization provides black-box solutions, in order to execute complex algorithmic tasks, but also an isolated environment that can be customized by the user. The proposed workflow, which is also distributed in containerized version, could have beneficial impact in the scientific community, providing a unified operator about metagenomics analysis and indicating a modern way to enhance repeatability in science and data analysis. Thus, the majority of comments concern to the core of metagenomics pipeline and not to the concept of virtualization.1. It would be useful to mention if there are other published containerized bioinformatics workflows (not only in metagenomics), or the proposed work is actually pioneering.2. In "Implementation" section, a justification for the choice of QIIME pipeline is important, as you sufficiently performed for all the other methodologies/tools. Also a short description about QIIME pipeline's functions that are used in YAMP is needed.3. "Input/Output" section may requires a clarification about how you define the set of default parameters (what reads length you used, how strict was the analysis, thresholds-errors tradeoff).4. You describe the disadvantages of other metagenomics analysis workflows, however there is not any comparison in "Results" section (time & resources consuming or quality control effectiveness). It would be notable to verify some of your introductory comments with a comparative analysis.

Level of Interest

Please indicate how interesting you found the manuscript: An article of importance in its field

Quality of Written English

Please indicate the quality of language in the manuscript: Acceptable

Declaration of Competing Interests

Please complete a declaration of competing interests, considering the following questions:

- Have you in the past five years received reimbursements, fees, funding, or salary from an organisation that may in any way gain or lose financially from the publication of this manuscript, either now or in the future?
- Do you hold any stocks or shares in an organisation that may in any way gain or lose financially from the publication of this manuscript, either now or in the future?
- Do you hold or are you currently applying for any patents relating to the content of the manuscript?

- Have you received reimbursements, fees, funding, or salary from an organization that holds or has applied for patents relating to the content of the manuscript?
- Do you have any other financial competing interests?
- Do you have any non-financial competing interests in relation to this paper?

If you can answer no to all of the above, write 'I declare that I have no competing interests' below. If your reply is yes to any, please give details below.

I declare that I have no competing interests

I agree to the open peer review policy of the journal. I understand that my name will be included on my report to the authors and, if the manuscript is accepted for publication, my named report including any attachments I upload will be posted on the website along with the authors' responses. I agree for my report to be made available under an Open Access Creative Commons CC-BY license (http://creativecommons.org/licenses/by/4.0/). I understand that any comments which I do not wish to be included in my named report can be included as confidential comments to the editors, which will not be published.

I agree to the open peer review policy of the journal

To further support our reviewers, we have joined with Publons, where you can gain additional credit to further highlight [illegible] work (see: https://publons.com/journal/530/gigascience). On publication of this paper, your review will be automatically [illegible] Publons, you can then choose whether or not to claim your Publons credit. I understand this statement.

Yes
